# Deletion of Polyamine Transport Protein PotD Exacerbates Virulence in Glaesserella (Haemophilus) parasuis in the Form of Non-biofilm-generated Bacteria in a Murine Acute Infection Model

**DOI:** 10.1080/21505594.2021.1878673

**Published:** 2021-02-02

**Authors:** Ke Dai, Zhen Yang, Xiaoyu Ma, Yung-Fu Chang, Sanjie Cao, Qin Zhao, Xiaobo Huang, Rui Wu, Yong Huang, Jing Xia, Qigui Yan, Xinfeng Han, Xiaoping Ma, Xintian Wen, Yiping Wen

**Affiliations:** a Research Center of Swine Disease, College of Veterinary Medicine, Sichuan Agricultural University, Chengdu, China; b Department of Population Medicine and Diagnostic Sciences, College of Veterinary Medicine, Cornell University, NY, USA

**Keywords:** *Haemophilus parasuis*, *glaesserella parasuis*, polyamine, potD, pathogenesis, apoptosis

## Abstract

Polyamines are small, polycationic molecules with a hydrocarbon backbone and multiple amino groups required for optimal cell growth. The *potD* gene, belonging to the ABC (ATP-binding cassette) transport system *potABCD*, encodes the bacterial substrate-binding subunit of the polyamine transport system, playing a pivotal role in bacterial metabolism and growth. The swine pathogen *Glaesserella parasuis* possesses an intact *pot* operon, and the studies presented here mainly examined the involvement of PotD in *Glaesserella* pathogenesis. A *potD*-deficient mutant was constructed using a virulent *G. parasuis* strain SC1401 by natural transformation; immuno-electron microscopy was used to identify the subcellular location of native PotD protein; an electron microscope was adopted to inspect biofilm and bacterial morphology; immunofluorescence technique was employed to study cellular adhesion, the levels of inflammation and apoptosis. The TSA++-pre-cultured mutant strain showed a significantly reduced adhesion capacity to PK-15 and MLE-12 cells. Likewise, we also found attenuation in virulence using murine models focusing on the clinical sign, H&E, and IFA for inflammation and apoptosis. However, when the mutant was grown in TSB++, virulence recovered to normal levels, along with a high level of radical oxygen species formation in the host. The expression of PotD could actively stimulate the production of ROS in Raw 264.7. Our data suggested that PotD from *G. parasuis* has a high binding potential to polyamine, and is essential for the full bacterial virulence within mouse models. However, the virulence of the *potD* mutant is highly dependent on its TSA++ culture conditions rather than on biofilm-formation.

## Introduction

*Haemophilus parasuis* (*H. parasuis*) is a nonmotile, pleomorphic, and nicotinamide adenine dinucleotide (NAD)-dependent bacterium which has been renamed in the NCBI taxonomy as *Glaesserella parasuis* under the family *Pasteurellaceae* [Dickerman et al., 2020]. It is one of the most common colonizers of the upper respiratory tract of swine and includes strains with diverse genetic and pathogenicity levels [Zhao et al., 2018a]. Multiple *G. parasuis* strains can exist in a single animal and, under certain conditions, virulent strain(s) can breach the mucosal barrier and enter the bloodstream, causing Glasser’s disease (GD) in piglets. Infected hosts typically display severe vascular lesions and multiple syndromes characterized by severe systemic inflammation accompanied by meningitis, pneumonia, polyarthritis, and fibrinous polyserositis. GD causes enormous economic losses in the global pork industry annually [Dai et al., 2019a; Pires Espindola et al., 2019]. Multiplex PCR (mPCR) method for rapid molecular typing of *G. parasuis* was used to confirm that *G. parasuis* comprises 15 serotypes plus several non-typeable (NT) isolates [Howell et al., 2015; Jia et al., 2017]. So far, 15 anti-microbial resistance genes have been found in this species [Kielstein et al., 1992; Spaic et al., 2019; Zhao et al., 2018b]. *G. parasuis* was primarily readily isolated in weaner pigs, followed by finisher pigs, and sows [Zhang et al., 2019]. A prerequisite for combating this illness to reduce the economic loss of GD is to determine the mechanism of its pathogenesis in-depth, to identify the virulence factors and/or discover new drugs to combat the infection in piglets and reduce the prevalence of anti-microbial resistance strains [de la Fuente et al., 2007].

Polyamines are a large class of polycationic biomolecules that exist in all living organisms. The three major polyamines, putrescine, spermidine, and spermine, are widely conserved among prokaryotic and eukaryotic cells [Thomas et al., 2001]. Polyamines are simple alkylamines with three or four low molecular weight amine groups naturally derived from arginine in cells that interact electrostatically with and stabilize negatively charged moieties, such as DNA, RNA, proteins, and phospholipids, thus participating in the regulation of multiple physiological functions. In microorganisms, polyamines are actively transported from periplasm to cytoplasm via a well-conserved ATP-binding cassette (ABC) transport system *potABCD*, which has been identified in many organisms [Kashiwagi et al., 1993]. Polyamine oligo-transport operon *potABCD* encodes a functionally highly relevant and specialized high-affinity transport system, wherein PotD serves as a spermidine/putrescine-binding protein, working as the direct binding receptor for both uptake and excretion of spermidine/putrescine. In contrast, PotA protein, an ATPase, binds ATP for spermidine uptake in conjunction with transport channels assembled by PotB and PotC, which anchors on the inner membrane [Furuchi et al., 1991; Yodsang et al., 2014]. It has been demonstrated using surface plasmon resonance (SPR) and radioisotope-labeling technology that PotD preferentially binds spermidine over putrescine in *Escherichia coli* and *Synechocystis* sp. [Brandt et al., 2010; Kashiwagi et al., 1996]. Five conserved polyamine-binding residues (Asp-Glu-Trp-Trp-Asp) with interestingly equal distances in different species have been found in PotD amino acid sequences, indicating conservation and similarity of function [Sugiyama et al., 1996a].

Currently, in addition to the ability of uptake and excretion of preferred polyamine, PotD protein was reported to be essential for the stimulation of SOS gene set expression and biofilm formation in *E. coli* [Zhang et al., 2013]. In *Legionella pneumophila*, deletion of *potD* gene can cause a series of puzzlingly complex phenotypes that include defects in *L. pneumophila*’s original capabilities to form filaments, associate with macrophages and amoeba, and to recruit host vesicles to *Legionella*-containing vacuoles (LCV), etc., suggesting that PotD is a multifunctional protein in this species [Nasrallah et al., 2014]. However, the exact biological functions of PotD and polyamines in *G. parasuis* remain unclear and have yet to be elucidated.

In previous studies, we and others have found that PotD is a stress-responsive protein and one of the differentially expressed virulence-related proteins in *G. parasuis* [Han et al., 2007; Zhang et al., 2014a]. We also found that recombinant PotD protein in *G. parasuis* could trigger an effectively immunoprotective effect against severe pathological lesions in a mouse model by triggering both cellular and humoral immunity [Dai et al., 2019b]. However, little evidence of the exact biological function of PotD, especially whether it is related to bacterial virulence *in vivo*, was unveiled in *G. parasuis*. In this study, we inactivated the *potD* gene in *G. parasuis* wild-type strain SC1401 and evaluated its tolerance for H_2_O_2_/oxidative stress and anionic detergent sodium dodecyl sulfate (SDS) by two different means of culture conditions. We also assessed its adhesion and invasion capability of non-biofilm-formation bacteria. We then investigated the virulence of the Δ*potD* mutant strain by assessing its reactive oxygen-inducing ability and inflammation/apoptosis levels in cellular and/or murine models. This study aims to determine the relationship between PotD and bacterial virulence of *G. parasuis*, thus providing better knowledge of connecting polyamine transport and mechanisms that underlay *Glaesserella* virulence levels.

## Materials and methods

### Animals

Adult female specific pathogen-free (SPF) BALB/c mice weighing 20–25 g (6-8-week-old) were purchased from Chengdu Dossy Experimental Animal Co., Ltd.

## Strains, Plasmids, Primers and Bacterial Growth Conditions

The bacterial strains and plasmids used in this study are listed in Table 1, and primers are listed in Table 2. *E. coli* DH5α and BL21(DE3) (Biomed, Beijing, China) were cultured in liquid Luria-Bertani (LB, BD-Difco, NJ, USA) medium or on LB agar (Invitrogen, Shanghai, China) plates. *G. parasuis* was grown in Tryptic Soy Broth (TSB, BD-Difco, NJ, USA) or on Tryptic Soy agar (TSA, BD-Difco, NJ, USA) plate supplemented with 5% inactivated bovine serum (Solarbio, Beijing, China) and 0.1% (w/v) nicotinamide adenine dinucleotide (NAD, Sigma-Aldrich, Maryland, USA) (TSB++ and TSA++). When necessary, the media were supplemented with 50 µl kanamycin (Kan, 100 mg/ml), 100 µl ampicillin (Amp, 100 mg/ml) or 100 µl gentamicin (Gm, 50 mg/ml). All strains grown in broth were cultured with shaking at 220 r/min (1 r = 2πrad) at 37°C, except where otherwise noted.

**Table 1.** Bacterial strains and plasmids used in this study

Strains or plasmids	Relevant characteristics	Sources or references
**Strains**		
*G. parasuis*		
SC1401	Serotype 11 clinical isolate (Sichuan, China), highly transformable strain	Laboratory collection
Δ*potD*::Kan	SC1401 derivative, *potD* deletion, Kan^R^	This study
Δ*potD*-c	SC1401 Δ*potD*::Kan complemented with pSF116-*potD*, Kan^R^Gm^R^	This study
1401D88	SC1401 derivative, point-mutation in *rpsL* gene, SM^R^	[Dai et al., 2018a]
*E. coli*		
DH5α	Standard *E. coli* cloning strain	Biomed
BL21(DE3)	Standard *E. coli* expression strain	Biomed
**Plasmids**		
pMD-19 T(simple)	Amp^R^, standard *E. coli* cloning T-vector	TaKaRa
pET-28a(+)	Kan^R^, standard *E. coli* expression vector	TaKaRa
pK18mobSacB	Kan^R^; suicide and narrow-broad-host vector	[Schafer et al., 1994]
pSF116	Gm^R^, *G. parasuis* complemented vector containing the 9-bp *G. parasuis* USS	[Zhou et al., 2016]
pET-*potD*	A 973-bp *potD* CDS without signal peptide sequence and termination codon in pET-28a(+)	This study
pK18-*potD*	Kan^R^, a 2732-bp fragment containing the USS and Δ*potD*::Kan cassette in pK18mobSacB	This study
pSF-c-*potD*	Kan^R^, a 1241-bp fragment containing the promoter region and CDS of *potD* gene in pK18mobSacB	This study
pKD4	Kan^R^, kanamycin resistance cassette-carrying vector	[Datsenko et al., 2000]

**Table 2.** Primers used in this study

Primers	Primer sequences (5ʹ→3ʹ)	products (bp)	Sources or references
**Conventional primers**			
P1 (HPS-F)	GTGATGAGGAAGGGTGGTGT	822	[He et al., 2018]
P2 (HPS-R)	GGCTTCGTCACCCTCTGT		
P3 (*potD*-F)	ATGAAAAAATGGGCTGTTGC	1041	This study
P4 (*potD*-R)	TTACTTCGCAGCTTTTAACTCT		
P5 (*potD*-pET-F)	cagcaaatgggtcgcggatccAATGACACTGTACATCTTTATACTT	1015	[Dai *et al*., 2019b]
P6 (*potD*-pET-R)	ctcgagtgcggccgcaagcttGCTTCGCAGCTTTTAACTCTTG		
P7 (*potD*L-F)	ctatgacatgattacgaattc**ACCGCTTGT**AGCTGAGTTTATGCCAGAGTTA	946	This study
P8 (*potD*L-R)	gcagggcttcccaaccttacCAATGTGTTCTCCTAAAAGAGT		
P9 (Kan-F)	GTAAGGTTGGGAAGCCCTGC	935	[Zhang et al., 2015]
P10 (Kan-R)	GGTCGGTCATTTCGAACCCC		
P11 (*potD*R-F)	ggggttcgaaatgaccgaccTTCATAGACATCGCTTAAAAAC	933	This study
P12 (*potD*R-R)	caggtcgactctagaggatccTCGCTAAATGTCACAATATTTC		
P13(pK18-F)	CTGGCACGACAGGTTTCC	342	This study
P14(pK18-R)	GCCTCTTCGCTATTACGC		
P15(*potD*-c-F)	gaagtttctatgtaaggtaccGTATTATCACCAAGATCAATTTG	1283	This study
P16(*potD*-c-R)	gcttatgtcaattcgggatccTTACTTCGCAGCTTTTAACTCT		
P17(pSF116-F)	GTTAACCCAGCTAACGTAACAG	308	This study
P18(pSF116-R)	GTCATAACAAGCCATGAAAACC		
P19(Gm-F)	ATGTTACGCAGCAGCAACGA	534	This study
P20(Gm-R)	TTAGGTGGCGGTACTTGGGTC		
**qPCR primers**			
P21(*lptA*-F)	GCAGCTAGATAGTGCCATTAAAGC	84	This study
P22(*lptA*-R)	TGTCGGTCATTACTCGTCGC		
P23(*lptC*-F)	AACAGTGGCAAGTCGAAGCC	102	This study
P24(*lptC*-R)	ACCGAGAAGTCGGATCTAAGC		
P25(*rng*-F)	GCAACACAGGCAATCGCACATC	101	This study
P26(*rng*-R)	ACACGCTGACAATGCTCTTCTTCC		
P27(*truA*-F)	TTGCCGTTATTGTGGGTGGC	131	This study
P28(*truA*-R)	GTACTCCCGCATCAGTTCGC		
P29(16s-F)	TGGTAGTCCACGCTGTAAAC	201	[Dai et al., 2018b]
P30(16s-R)	AGGATGTCAAGAGTAGGTAAGG		

Lowercase letters are homologous recombination fragments required for in-fusion clone to the adjoining segments or vectors. *G. parasuis* uptake signal sequence (USS) used to promote natural transformation is underlined with bold lines.

## Expression/purification of recombinant potd protein, and preparation of mouse antiserum

The expression of the recombinant protein of PotD (rPotD) was generated using an *E. coli* expression system. Briefly, PCR fragments containing the *G. parasuis potD* gene (– its 22 amino acid signal peptide sequence) were amplified from the genomic DNA of SC1401 using primers P5+ P6 (*potD*-pET-F/R, Table 2) (2× Fast Pfu Master Mix, Novoprotein). The resulting 1015-bp PCR products were purified (HiPure PCR Pure Mini Kit, Magen) and cloned into restriction enzyme sites BamHI and HindI of linearized pET-28a(+) using ClonExpressII One Step Cloning Kit (Vazyme, Jiangsu, China), giving rise to pET-*potD*. At an optical density at 600 nm (OD_600_) of 0.5 to 0.6, *E. coli* BL21(DE3) bearing pET-*potD* was induced to express by the addition of 1 mM isopropyl-β-D-thiogalactopyranoside (IPTG) for 5 h. The culture was spun at 5,000 × g for 10 min, resuspended in 50 mM Tris-HCl, and then lysed by sonication. The lysate was further centrifuged at 12,000 × g for 10 min to pellet cell debris. The His-tag fusion recombinant protein was purified by Ni affinity chromatography using a Bio-Scale^TM^ Mini Nuvia^TM^ IMAC Ni-Charged Resin (5 ml prepacked column, Bio-Rad). Purified rPotD was dialyzed with 2 L of PBS for 2 d and then analyzed by SDS-PAGE electrophoresis. After being concentrated by a 10-Kd ultrafiltration device (Millipore, USA), the concentration of recombinant protein was determined using a Modified BCA Protein Assay Kit (Sangon Biotech, Shanghai, China). A ToxinEraser^TM^ Endotoxin Removal Resin (Genscript, NY, USA) was used to treat the purified protein by reducing endotoxin contamination to lower than 0.1 EU/ml [Banerjee et al., 2011]. Chromogenic End-point LAL assay was adopted to quantify the endotoxin as previously described [Zhang et al., 2018].

The anti-rPotD antisera were prepared as previously described [Dai *et al*., 2019b]. Each mouse was immunized subcutaneously on days 0, 14, and 21 with 0.1 mg of rPotD (0.2 ml) containing a one-tenth volume of water adjuvant MONTANIDE™ GEL 01 (SEPPIC, Paris, France) [Xu et al., 2019]. Blood was collected using silica capillaries and non-additive vacuum blood tubes by retro-orbital bleeding on day 28. Serum was naturally precipitated after incubation at 4°C for half an hour, and then the mixture was centrifuged at 10,000 × g for 10 min to separate the serum further.

A standard indirect Enzyme-linked immunosorbent assay (ELISA) using a purified rPotD protein-coated polystyrene microtiter 96-well plate (Costar; USA) and twofold serially diluted PotD antiserum in a range of 10^−2^-10^−5^ was used to determine the neutralizing antibody IgG titers of the serum samples [Nieto et al., 2015]. The titer was defined as the absorbance of the highest dilution at a wavelength of 450 nm (A_450nm_) whose value was at least 0.1 above the average value of the background wells in which PBS replaced serum samples. The experiments were independently performed at least three times in triplicate.

## Construction and complementation of the *potd* mutant and western-blotting confirmation

To construct an in-frame nonpolar *potD* mutant, the target vector pK18-*potD* was built as follows: first, the 946-bp encompassing the upstream homologous region and the 933-bp downstream homologous region of the in-situ *potD* gene were amplified using primers P7+ P8 (*potD*L-F/R, Table 2) and P11+ P12 (*potD*R-F/R) from the genomic DNA of *G. parasuis* SC1401 (prepared by a TIANamp Bacteria DNA Kit, TIANGEN, Beijing, China), then the 935-bp kanamycin-resistant cassette (Kan^R^, *aminoglycoside phosphotransferase Aph(3ʹ)-IIa* gene) was amplified by PCR from pKD4 using primers P9+ P10 (Kan-F/R). Subsequently, these three fragments were purified using a Qiaquick spin column kit (Qiagen, Hilden, Germany) and were ligated into a linearized vector pK18mobSacB, which was digested by EcoRI and BamHI using a BM seamless cloning kit (Biomed, Beijing, China). Finally, the resulting plasmid pK18-*potD* was transformed into *G. parasuis* SC1401 using a natural transformation technique. An optimized protocol for natural transformation was performed as previously described [Dai *et al*., 2018b; Zhang et al., 2014b]. In particular, one copy of the 9-bp DNA uptake signal sequence (USS) of 5ʹ-ACCGCTTGT was added to the forward primer of the upstream homologous fragment (P7) for the sake of elevating natural transformation. Transformant bacteria were incubated for 36 h. The resultant kanamycin-resistant transformants were cultured at a large scale in TSB++/Kan for further identification by PCR (using P3+ P4 for *potD* deletion, and P9+ P10 for the presence of Kan cassette) and Western blotting (WB). To rule out possible polarity effects resulting from *potD* gene deletion, relative quantification of 2(–ΔΔC(T)) method was adopted to analyze the flanking genes’ transcriptional levels by quantitative real-time PCR (qRT-PCR) using primer sets P21/P22 for *lptA* (lipopolysaccharide export system protein), P23/24 for *lptC* (lipopolysaccharide export system protein), P25/P26 for *rng* (ribonuclease G) and P27/P28 for *truA* (tRNA pseudouridine synthase A), respectively. Here, the stably transcriptional 16S RNA of *G. parasuis* was used as an internal reference gene. qRT-PCR was performed by using an iTaq^TM^ universal SYBR Green Supermix (Bio-Rad) in a Lightcycler96 (Roche, Switzerland) system.

For genetic complementation, the 200-bp promoter and entire coding sequence (CDS) of *potD* together were cloned into a *G. parasuis* complemented vector pSF116 at sites KpnI and BamHI to generate plasmid pSF-c-*potD*, which was verified by primers P17+ P18. The resulting plasmids were naturally transformed onto the chromosome of *potD* in-situ mutant Δ*potD*::Kan in maximal competence-inducing conditions to get the *potD* ectopic complemented strain Δ*potD*-c. Transformants were selected on TSA++/Gm (12.5 mg/ml). The complemented strain was further confirmed by PCR (using P3+ P4 for *potD* reappearance and P19+ P20 for the presence of Gm cassette) and WB.

For WB identification of the successful constructs of Δ*potD***::**Kan and Δ*potD*-c, the whole extract of candidate *potD* mutant and complemented strains were analyzed using the PotD antiserum prepared as above. Briefly, the whole-cell lysates of both strains and wild-type SC1401 (control) were analyzed by 12% SDS-PAGE and electrotransferred onto a 0.22 μm PVDF membrane. After being activated in methanol for 30 s and blocked with 5% skim milk in TBST at room temperature (RT) for 30–60 min, the membrane was washed five times and incubated at RT for 1 h or at 4°C overnight with mouse antiserum against rPotD as the primary antibody. The membrane was rinsed 3–5 times with no less than 25 ml of TBST per wash. Secondary antibody (Rabbit-anti-mouse, 1: 10,000 dil.) conjugated with horseradish peroxidase (HRP) was added to the whole PVDF membrane in 15 ml of TBST. The membrane was finally treated with chemiluminescence reagent ECL (Beyotime). The grayscale bands were developed by the ChemiDoc^TM^ XRS+ system with Image Lab^TM^ software (Bio-Rad).

## Immuno-electron microscopy (IEM) Inspection of Native PotD Protein in *G. parasuis*

The subcellular location of native PotD protein in bacteria was performed by using immuno-electron microscopy (IEM) technology. Samples (SC1401 and derivatives Δ*potD*::Kan) for IEM were prepared as follows. Overnight-grown suspended bacterial culture was spun down and pellets were resuspended in electron microscope fixing solution (2.5% glutaraldehyde; Goodbio technology CO. LTD, Wuhan, China) for 2 h in the dark. Ultrathin sections were prepared as previously described [Dai *et al*., 2019b], the samples were washed, blocked, and incubated with primary antibody (mice-sourced PotD antisera). Secondary antibody (Sigma), which was chelated with colloidal gold particles, was added, and the sections were incubated for 1 h at 37°C. Finally, the sections were further counterstained by 2% uranium acetate and saturated alcohol solution for 8 min, and washed three times by 70% alcohol, followed by three washes using ultrapure water. The sections were dried and visualized by using TEM (HITACHI, HT7700, Japan). The 10 nm nanogold particles were recognized as colocalization of native PotD.

## Genetic stability and growth characteristic of the mutant strain Δ*potD***::**Kan

To rule out that virulence attenuation or enhancement resulting from the altered metabolic level of Δ*potD***::**Kan, and/or reverse mutation, we further assessed the growth characteristic and genetic stability of the mutant strain. Briefly, the wild-type *G. parasuis* SC1401 and derivatives Δ*potD***::**Kan and Δ*potD*-c were inoculated and propagated with agitation in TSB++ repeatedly at an interval of 12 h per generation. All cultures were continuously passaged for 20 generations. In each subculture step, the in vitro growth kinetics of the tested strains were determined by monitoring the optical density 600 nm (OD_600nm_) with a spectrophotometer (Smartspec^TM^ Plus, Bio-Rad). At the same time points, viable colonies of bacteria were quantified by plating serial dilutions (10^−6^ and 10^−7^) onto TSA++ plates. The experiments were independently performed at least three times in triplicate. The stabilities of *potD* gene in both the mutant and complemented strains were determined using PCR up to their respective 20th generation.

## Autosedimentation assay

The ability of *G. parasuis* to autoagglutinate was evaluated as previously described with some modifications [Zhang et al., 2016]. *G. parasuis* SC1401 and derivatives grown on TSA++ plates were inoculated into 3 ml of TSB++ and cultivated at 37°C overnight. The adherent and planktonic cells were harvested and diluted in sterilized PBS (pH = 7.4) to an OD_600nm_ of 0.8 and then allowed to remain static in disposable glass cuvettes at RT. The OD_600nm_ values of the suspensions were measured using a spectrophotometer (Smartspec^TM^ Plus, Bio-Rad) every 1 h for 24 h.

## Biofilm formation, scanning electron microscope and transmission electron microscope

The ability of *G. parasuis* strains to form biofilms in TSB++-cultured condition was quantified by the crystal violet incorporation assay with modifications [Liu et al., 2018]. Briefly, *G. parasuis* strains SC1401 and derivatives Δ*potD*::Kan and Δ*potD*-c were cultured in TSB++ medium to an OD_600nm_ reaching 0.6–0.8 and then normalized to 0.1 (about 1.25 × 10^8^ CFU/ml). A ten microliter aliquot of bacteria was inoculated into 2 mL TSB++ medium, which was incubated at 37°C with circular agitation (80 rpm/min) for 24 h. The content in the tubes was then removed with an injector. Fresh TSB++ medium was added to another tube as a blank control. Next, the tubes were washed three times with sterile PBS to remove loosely adherent cells. After washing, each tube was fixed with 2 ml of 99% (v/v) methanol for 15 min and allowed to air dry at RT. After that, each tube was stained with 2 ml of a 1% Hucker crystal violet solution at RT for 10 min. Unbound dyestuff was subsequently removed with an injector and washed under running water for 3–5 min. Finally, the bound crystal violet was dissolved from each tube by using 2 ml of 95% ethanol for 10 min. The absorbance at 570 nm (OD_570nm_) was measured. All assays were performed three times in triplicate.

Samples (SC1401 and derivatives Δ*potD*::Kan) for SEM and TEM were prepared as follows. For statically cultured bacteria, multiple colonies of each sample plus their agar were scraped off and put in electron microscope fixing solution (2.5% glutaraldehyde; Goodbio technology CO. LTD, Wuhan, China) for 2 h in the dark. For suspended bacterium culture, samples were spun down; pellets added to the electron microscope fixing solution and fixed as above. After sections were prepared as previously described [Dai *et al*., 2019b], the samples were visualized using SEM (HITACHI, SU8100, Japan) and TEM (HITACHI, HT7700, Japan), respectively.

## Oxidative stress tolerance and SDS sensitivity assays

Lung and the upper respiratory tract, as a natural niche for *G. parasuis* to colonize and proliferate, are usually rich in reactive oxygen species (ROS), which is detrimental to the survival of this bacterium. High levels of ROS produced by macrophages through a respiratory burst is one mechanism the immune system uses to protect the host from pathogens. To explore their different oxygen resistance abilities, for statically cultured bacteria, we mimicked the attack of a high concentration of oxygen in vitro using different concentrations of H_2_O_2_ and Oxford cups. Briefly, 200 μl bacterial suspension (1.0 × 10^7^ CFU/ml) was spread evenly over a TSA++ plate. Afterward, one hundred microliters of different concentrations (50 mM to 200 mM) of H_2_O_2_ were pipetted to the Oxford cups, which were placed carefully on the agar surfaces. The diameter of the inhibitory ring for each concentration was measured after a 16-h incubation at 37°C. For bacterial culture, the newly incubated bacteria, which had been grown at 37°C with circular agitation (80 rpm/min) for 24 h, were exposed to different final concentrations of H_2_O_2_ (0–64 mM) at aliquots of 200 µl at 37°C for 30 min. Viable colonies of bacteria were quantified by plating serial dilutions (10^−2^-10^−5^) onto TSA++ plates. The experiments were independently performed at least three times in triplicate.

To determine if the observed altered sensitivity mediated by biofilm was common to other membrane-active agents, we tested the sensitivity of both biofilm-producing strains to the anionic detergent SDS. Assays on the inhibitory effect of SDS were carried out as above except for the working concentration utilized in both experiments (plate- and broth-cultured bacteria).

## Cell culture, invasion and adhesion assays

Adhesion and invasion assays were performed to investigate possible reduced infection abilities of planktonic Δ*potD*::Kan on two epithelial cell lines PK-15 (ATCC-sourced strain, CCL-33) and MLE-12. Swine kidney cell line PK-15 was maintained in complete Dulbecco’s modified Eagle’s medium (DMEM) supplemented with 10% (v/v) fetal bovine serum (FBS) and penicillin-streptomycin (PS; 100 IU/ml and 100 µg/ml, respectively; Gibco, Carlsbad, CA). Mouse lung epithelial cell line MLE-12 was purchased from the Beijing Institute of BeNa Biotechnology (ATCC-sourced strain, the 4th passage). MLE-12 cells were cultured in a 1:1 mixture of DMEM and Ham’s F-12 nutrient mixture (DME/F12; Hyclone, Logan, UT), supplemented with 2.5 mM L-glutamine and 15 mM HEPES buffer, 10% (v/v) fetal bovine serum (FBS) and penicillin-streptomycin (PS; 100 IU/ml and 100 µg/ml, respectively; Gibco, Carlsbad, CA). Cells suspensions (about 5 × 10^5^ cells/ml) were seeded into 6-well tissue culture plates and were cultured at 37°C in a humidified incubator with 5% CO_2_ for 12 h before use.

For adhesion assay, the wild-type *G. parasuis* SC1401, derivatives Δ*potD*::Kan and Δ*potD*-c were grown to logarithmic growth. The planktonic bacteria, rather than biofilm-formed bacteria, were transferred to new centrifuge tubes. These bacteria were washed in PBS by centrifugation, resuspended in cell culture medium and adjusted by dilution to provide an MOI of 10:1 bacteria to host cells in culture wells of a six-well plate (about 1 × 10^6^ host cells vs. 1 × 10^7^ bacteria). Confluent monolayers were infected for 2 h at 37°C to allow for bacterial adhesion. After incubation, cells were rigorously washed three times with sterile PBS to eliminate nonspecific bacterial attachment and were then incubated for 10 min at 37°C with 100 µl of 0.25% trypsin/EDTA. After incubation, 900 µl of ice-cold TSB was added, the cells were removed from the culture plates by scraping the bottoms of the wells. Bacterial enumeration was performed using serial 10-fold dilutions and plating on TSA++ plates. Bacteria were incubated overnight at 37°C, and the visible colonies were counted manually to calculate the CFU.

For the invasion assay, bacterial preparation, PK-15/MLE-12 cells culture, bacterial infection, and counting were performed as described above except that the extracellular bacteria were killed by incubation of the monolayer with DMEM containing chloromycetin (25 µg/ml) for another 2 h following the incubation with the bacteria and three washes with PBS. All the experiments were independently performed at least three times in triplicate.

## Indirect immunofluorescence assay of adherent bacteria on MLE-12

In IIF assay, MLE-12 cells were grown on coverslips in a 6-well plate to reach a cell concentration of 90%. After bacteria and cells were prepared and treated as following the adhesion assay procedure, IIF assays were conducted to detect the distribution of bacteria by mouse-derived HpGbpA polyclonal antibody (1:500 dil.; unpublished) and Cy3-conjugated goat-anti-mouse IgG (Servicebio). Photos were taken using a fluorescence microscope (Nikon Eclipse ci&NIKON DS-U3) to detect Cy3-labeled fluorescent secondary antibodies (EX WL: 510–560 nm, EM WL: 590 nm) and analyzed by an immunofluorescence assay using the CaseViewer software. Fluorescence signal intensities were shown as integrated optical density (IOD) values, analyzed by ImageProPlus software. Relative values of IOD_R_ were calculated by the ratio of the IOD of Cy3-fluorescence intensity (IOD_C_) and DAPI (IOD_D_).

## Cellular and tissular ROS measurement

Pathological lesions of PotD protein were primarily evaluated by testing the production of intracellular ROS in a mouse monocyte-macrophage leukemia cell line RAW 264.7. Intracellular ROS level was determined by detecting the changes of fluorescence intensity resulting from the oxidation of the fluorescent probe dichlorodihydrofluorescein diacetate (DCFH-DA; Sigma-Aldrich, Milan, Italy). In brief, RAW 264.7 cells (2 × 10^5^ cells/ml) were seeded in 12-well plates and incubated for 12 h. Then, the confluent monolayers of cells were exposed to purified rPotD (50 μg/ml), LPS (1 μg/ml, positive control) or TSB++ (negative control) for 16 h. Next, the cells were washed, counted, and incubated withDCFH-DA (20 μM) in a serum-free medium for 30 min at 37°C in the dark. Subsequently, cells were washed again and resuspended in PBS, and the oxidative formation of DCFH-DA in RAW 264.7 cells via intracellular ROS, expressed as mean fluorescence intensity (MFI), was immediately examined using a flow cytometer (FACSVerse, BD Biosciences, San Jose, CA, USA) using a 488 nm excitation filter and a 525 nm emission filter (similar to the FITC optical path). All experiments were performed in triplicate with at least 10,000 cell counts per treatment. The frequencies of ROS-positive cells were analyzed by Flowjo software (Flowjo LLC, Ashland, OR, USA). The experiments were independently performed at least three times in triplicate.

In order to further evaluate the level of ROS directly in vivo, we applied a frozen section and immunofluorescence assay (IFA) for myocardia and spleen tissues of the mice challenged with wild-type G. *parasuis* SC1401 and its derivatives Δ*potD*::Kan, and Δ*potD*-c after 4 dpi. Section preparation and protocol for IFA are described as follows: In particular, sections were incubated with dihydroethidium (DHE), and slides were counterstained with 4′,6-diamidino-2-phenylindole (DAPI), and photos were captured. Ethylene oxide integrates into chromosome DNA, producing violet fluorescence to serve as a ROS reference intensity. Relative values of IOD_R_ were calculated by the ratio of the IOD of fluorescence intensity (IOD) and DAPI (IOD_D_), analyzed by ImageProPlus software. The experiments were independently performed at least three times in triplicate.

## Serum bactericidal assays

The serum bactericidal assay was performed as described previously [Ding et al., 2016]. Briefly, porcine serum was obtained from healthy piglets (3-4-weeks-old) with no history of *G. parasuis* infection, further confirmed by a *G. parasuis* antibody test kit (BIOVET, Saint-Hyacinthe, QC, Canada). The serum was filter-sterilized (0.22 µm) before use. SC1401, Δ*potD*::Kan, and Δ*potD*-c were cultured in TSB++ to reach an OD_600nm_ of 0.8. Some aliquots of the sera were treated at 56°C for 30 min to inactivate complement, serving as a negative control. Then, each aliquot of fresh porcine serum or heat-treated porcine serum was mixed with planktonic bacteria to obtain three final different concentrations (10%, 20%, or 50%) of serum. The mixtures were incubated at 37°C for 1 h with gentle agitation (130 rpm/min). Subsequently, the mixtures were 10-fold serially diluted and spread on TSA++ plates. The plates were incubated at 37°C for 24 h, after which the colonies were counted manually. The survival rates were calculated by determining the ratio of colonies in the fresh serum to those in the heat-treated serum.

## Animal infection studies

To assess the virulence of plate-cultured and broth-cultured bacteria, 60 mice were allocated randomly to SC1401, Δ*potD*::Kan, and Δ*potD*-c groups (G1-G3 for plate-cultured cells and T1-T3 for broth-cultured cells, each group contained 10 mice), and another 10 mice were allocated to PBS control group (M, mock group). All mice were simultaneously intraperitoneally injected with 1.3 × 10^9^ CFU/mouse (0.5 ml) according to the approved animal care protocol, as stated above, and in strict accordance with the regulations related to an operating procedure for laboratory animals, Procedure With Care (www.procedureswithcare.org.uk/subcutaneous-injection-in-the-rat). Virulence was evaluated daily by carefully monitoring clinical symptoms and life-or-death status for 7 d. The surviving mice were euthanized 4 d post-infection (dpi). Clinical signs were determined according to murine activity scores, which were recorded by the sum of the following grading standards: poor spirit (1 point), loss of appetite (1 point), hunched posture (1 point), increased heart rate (1 point), fur loss (1 point), and weight loss (1 point). Mice showing more severe symptoms received higher scores [Wang et al., 2019].

## Histological examination (H&E) and immunofluorescence (IF) assay

Mice were euthanized using sodium pentobarbitone. Lung and spleen tissues were harvested from mice in different groups (G1-G3, T1-T3, and M) after 4 dpi and fixed with 4% paraformaldehyde for histopathological and immunofluorescence (IF) assay. Several sections (4–5 mm thick) embedded in paraffin were prepared, stained with hematoxylin and eosin (H&E) for histopathological examination. Pathophysiological structures of 5–8 μm sections were visualized by light microscopy (Nikon Eclipse ci) and analyzed by Nikon digital sight DS-F12 (Japan) [Quan et al., 2018]. Pathological lesions were evaluated by the histopathological alterations, which were recorded by the following grading standards: mild (1 point), minor (2 points), moderate (3 points), severe (4 points), extremely serious (5 points), asymptomatic (0 points). The evaluation contents covered the presence of inflammation, type(s) of cellular infiltration (hemorrhage, extravasated blood), and pathological changes (infarct, necrosis) [Guarner et al., 2001; Mateus et al., 2019].

Immunofluorescence assay for myeloperoxidase levels (MPO) was performed to further quantitatively investigate the levels of inflammation in the lungs and spleens of the mice challenged with wild-type *G. parasuis* SC1401 and its derivatives Δ*potD*::Kan, and Δ*potD*-c after 4 dpi. Mouse tissue and sections preparation were similar to that of H&E material. Formalin/paraformaldehyde-fixed, paraffin-embedded (FFPE) tissue sections of 5–8 µm thickness were deparaffinized and rehydrated. In a closely following epitope retrieval step, sections were heated at 85–90°C for 8 min, set aside for 8 min at RT, followed by heating at 50–60°C for another 8 min in antigen repair buffer EDTA (pH = 6.0). Slides were allowed to cool to RT and then washed three times in PBS (PH = 7.4) for 5 min each. Sections were treated with AutoFluo Quencher (Sevicebio, Wuhan, China) for 5 min and then blocked with BSA for 30 min at RT. After a gentle rinse in running water for 10 min, sections were incubated with primary antibody diluted in PBS with 0.05% Tween-20 overnight at 4°C in humidifying boxes. Sections were then washed in PBS (PH = 7.4) 3 times for 5 min each, and sections were incubated with secondary antibody in PBS-Tween for 50 min at RT in the dark. After being washed again in PBS (PH = 7.4), sections were counterstained with DAPI for 10 min at RT in the dark for another wash. All sections were mounted using an Antifade Mounting Medium (Beyotime). Photos were shot and analyzed as above. Relative values of IOD_R_ were calculated by the ratio of the IOD of Cy3-fluorescence intensity (IOD_C_) and DAPI (IOD_D_), analyzed by ImageProPlus software. The experiments were independently performed at least three times in triplicate.

## Apoptosis assay (TUNEL staining)

For further observation of cell apoptosis in the lung and spleen tissues of mice in situ affected by wild-type *G. parasuis* SC1401 and its derivatives Δ*potD*::Kan, and Δ*potD*-c after 4 dpi., we performed TUNEL (TdT-mediated dUTP nick end labeling) analysis based on histopathological and IF techniques. Sections of challenged lungs and spleens of mice from G1-G3/T1-T3 and M (mock group) were routinely prepared as stated above. After being deparaffinized (as stated above), antigen retrieval (Proteinase K, 30 min, 37°C) treated, permeabilized (0.2% TritonX-100, 20 min, RT), Sections were treated to a TUNEL staining, as per the manufacture’s specification of an in situ Cell Death Detection kit, POD kit (Roche, Switzerland). All fluorescent images were examined via fluorescence microscopy and photographed to detect fluorescein isothiocyanate (FITC)-labeled fluorescent secondary antibodies (EX WL: 465–495 nm, EM WL: 515–555 nm). Immunofluorescence assay was performed using the CaseViewer software. The absolute values of fluorescence signal intensity were shown as integrated IOD values, analyzed by ImageProPlus software. Relative values of IOD_R_ were calculated by the ratio of the IOD of FITC-fluorescence intensity (IOD_F_) and DAPI (IOD_D_).

## Bioinformatics analysis, image and statistical analysis

Nucleotide sequences of *pot* operons *potABCD* of *G. parasuis* and other polymicrobial nucleotide sequences were retrieved from Genbank. These included *G. parasuis, Actinobacillus pleuropneumoniae, Haemophilus influenzae, Escherichia coli, Streptococcus pneumoniae*, etc. The whole amino acid sequences of the CDSs were translated using ORFfinder (https://www.ncbi.nlm.nih.gov/orffinder/). Sequence distances among different microbes were analyzed using the MegAlign program in Mega6 software (Neighbor-joining method, NJ). Molecular structures of spermidine were retrieved from PubChem Compound database (PubChem CID: 1102). Crystal structure of *E. coli* was downloaded from Protein Data Bank (PDB: 1POT). *Synechocystis* sp., *L. pneumophila,* and *G. parasuis* PotD were modeled based on the real X-ray structure of *E. coli* PotD and the alignment using the program server Phyre2 [Kelley et al., 2015]. Protein tertiary structures were displayed by programs PyMol and Chimera. Molecular docking was performed using SDF file of spermidine and PDB file of the spermidine-binding domain in PyMol. A set of nine models was created with the spermidine and its ligand in the active site. Phylogenetic trees were pruned using the online server iTOL v4 (https://itol.embl.de/). Conserved polyamine-binding residues and consensus between several strains were drawn using software Jalview 2.10.3.

Comparisons of several independent test series were evaluated by analyzing two-way analysis of variance (ANOVA tests). Multiple comparisons between any two means of different groups were performed using the least significant difference (LSD) method. Values were presented as mean values plus or minus the standard error of the mean (S.E.M.). A p-value was generated for all variables. A p < 0.05 was considered to be statistically significant (*).

## Results

### G. parasuis *PotD bears Polyamine-binding Residues and is Conserved among* G. parasuis

We used BLAST and SC1401 *potD* sequence to query the genome sequences of all *G. parasuis* deposited at GenBank and found *potD* to be a single-copy gene, which facilitated gene-mutation construction. The whole CDS of *potD* is 1,041 bp, encoding 346 amino acid residues, which equals 38.45 kDa in relative molecular mass, predicted by the ExPASy ProtParam tool. The theoretical pI is 5.18, suggesting that under a conventional physiological environment in situ, PotD protein is negatively charged, an opposite charge against polyamines, consisting primarily of three positively charged small molecules: putrescine, spermidine, and spermine. These compounds exist as linear molecules with 2–4 positively charged nitrogen atoms [Sugiyama et al., 1996b]. This may underscore one possible physical way for them to bind each other through ionic bond formation, as reported that polyamines interact with various macromolecules both electrostatically and covalently in a variety of species [Wallace et al., 2003]. Structural and functional studies have established that polyamines are in a fully protonated state under physiological conditions, thus are positively charged and usually interact with intracellular negatively charged compounds, such as nucleic acids, ATP, and phospholipids [Childs et al., 2003; Miyamoto et al., 1993].

**Figure 1.**
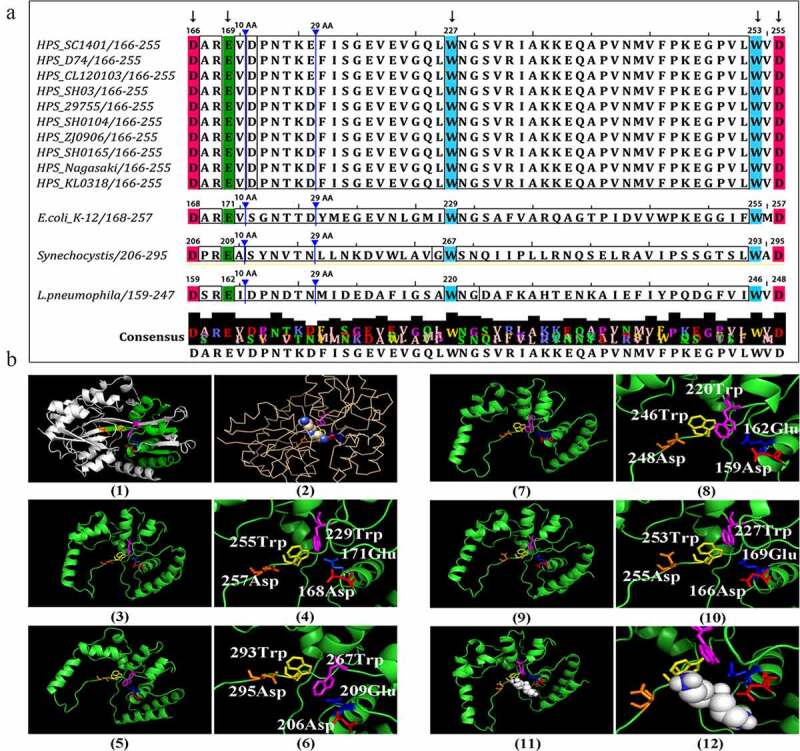
**Putative polyamine-binding residues in *G. parasuis* PotD amino acid sequences and homology models of PotD proteins and their molecular dockings to spermidine**. (a) The conservation of the five polyamine-binding residues Asp-Glu-Trp-Trp-Asp in PotD at an equivalent distance among different strains was also presented in all *G. parasuis* strains, which was concordant for the well-proved polyamine-binding residues/sites (black arrows) in *E. coli, L. pneumophila* and *Synechocystis* sp. strain PCC 6803. Sequence consensus indicated that besides the five conserved polyamine-binding residues, other residues were more variable especially among different species. (b) Homology models of PotD proteins and their molecular dockings to spermidine. **(1)** The overall structure of *E. coli* PotD; **(2)** simplified tertiary structure of *E. coli* PotD with bound spermidine; central cleft regions of the polyamine-binding domain from *E. coli* **(3)**, *Synechocystis* sp. **(5)**, *L. pneumophila* **(7)** and *G. parasuis* **(9)**; enlarged drawings and corresponding spermidine-binding amino acid residues in *E. coli* **(4)**, *Synechocystis* sp. **(6)**, *L. pneumophila* **(8)** and *G. parasuis* **(10). (11)** and **(12)** possible molecular docking of spermidine and spermidine-binding domain in *G. parasuis* PotD. The conservation of the five residues Asp-Glu-Trp-Trp-Asp in PotD protein at an equivalent distance among different strains were presented using different colors in the core region of the most likely polyamine-binding domain. One possible patterns graph of the tertiary structure of spermidine was shown in spheres, whose molecular formula was depicted in **Figure S1.**

The crystal structure of PotD has been identified using X-ray by other researchers, serving as a potent three-dimensional homologous modeling template to determine spatial structures and physicochemical properties of binding polyamine(s) [Sugiyama *et al*., 1996a; Sugiyama *et al*., 1996b]. The PotD protein of *E. Coli* consists of two domains with an alternating beta-alpha-beta topology (Figure 1b). The polyamine binding sites are in a central cleft lying in the interface between the domains, where beta-pleated sheets are mainly presented. In the cleft, four acidic residues recognize the three positively charged nitrogen atoms of spermidine, while five aromatic side chains anchor the methylene backbone by Van der Waals interactions. Like several other substrate-binding subunits of colibacillary and cyanobacterial ABC transporters, investigation against five conserved polyamine-binding residues in *G. parasuis* PotD amino acid sequence showed that it bears coincident ligand-binding sites, which was concordant for the well-established polyamine-binding residues/sites in *E. coli, L. pneumophila* and *Synechocystis* sp. strain PCC 6803 at an exactly equidistant amino acid residue [Brandt *et al*., 2010; Nasrallah *et al*., 2014; Sugiyama *et al*., 1996b] (Figure 1a). The conservation of the five polyamine-binding residues was Asp-Glu-Trp-Trp-Asp, whose side chains were drawn codirectionally toward spermidine in the core region of PotD (Figure 1b), forming a more compact “pocket-like” structure by comparison to that of two maltodextrin-binding protein structures from *E. coli* [Sugiyama et al., 1996b]. Moreover, we also found amino acid residues proline (P, site 246 in *G. parasuis*) and glycine (G, site 249) were also conserved in the strains we blasted (Figure 1a), which had not been reported. It intrigued us to connect these two sites to spermidine-binding ability, for example, by using the surface plasmon resonance (SPR) technique and site-directed mutation. Through molecular docking of spermidine and spermidine-binding domain in *G. parasuis* PotD, we speculated nine possible models for the spermidine and its ligand in the active site with a range of affinities between −3.1~-0.5 kcal/mol (**Figure S1**). Together, it is tempting and logical to speculate that *G. parasuis* can actively bind to polyamine, especially the spermidine. Elucidating this similar structure will provide valuable insight into its physiological role in the binding and uptake of polyamines in vivo.

**Figure 2.**
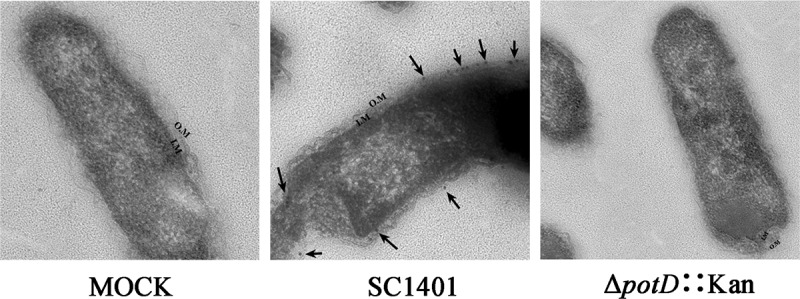
**Immunoelectron microscopy (IEM) inspection of subcellular localization of native PotD protein in *G. parasuis*** (**×30k**). The nanogold particles indicating the original location of native PotD protein were marked with arrows. O.M: outer membrane; I.M: inner membrane

In other physicochemical characteristics, the instability index (II) was computed to be 30.65, classifying the protein as stable. The grand average of hydropathicity (GRAVY) is −0.331, showing that PotD is likely soluble in water (hydrophilic). No transmembrane helix was predicted (TMHMM Server v. 2.0). By Gneg-mPLoc server, we predicted that PotD is located in periplasm, and this was visually verified by immuno-electron microscope (IEM) technology for the first time in this work (Figure 2).

**Figure 3.**
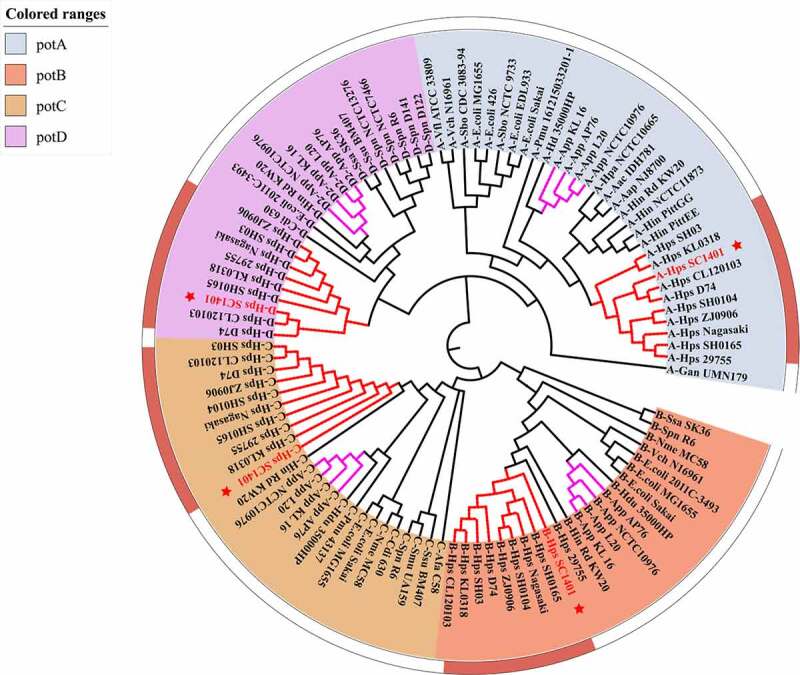
**Phylogenetic tree of *potABCD***. Neighbor-joining (NJ) tree demonstrated the overall diversity of PotDs from *H. parasuis* (*Hps, G. parasuis), A. pleuropneumonia* and other pathogens. The *potABCD* operon homologs can be grouped into four major clades corresponding to the functionally highly connected and specialized high-affinity transport system modules *potA, potB, potC* and *potD*. Genes were labeled as “gene name-species name plus strain name” listed in Supplementary Table S1. Gene clusters of interest to this study are labeled in red

Phylogenetic analysis demonstrated that the *potABCD* gene cluster was widespread in several representative species of bacteria, including *H. influenzae, A. pleuropneumonia, E. coli, G. parasuis, S. pneumoniae*, etc (Figure 3). Blast analysis showed that HpPotD belonged to one sole clade, suggesting that this protein is highly conserved in *G. parasuis*, which endows this protein with a potential capacity to serve as a good candidate for a subunit vaccine against this species. In contrast, further analysis revealed that the sequences used in this study clustered into different main groups according to their corresponding species, implying that the host specificity has evolved over relatively long periods. From this point of view, it would be logical to speculate PotD plays a different role or participates in complex functions in heterologous species, as reported in numerous studies.

## Purification of rPotD protein, endotoxin elimination and polyclonal antibody preparation

**Figure 4.**
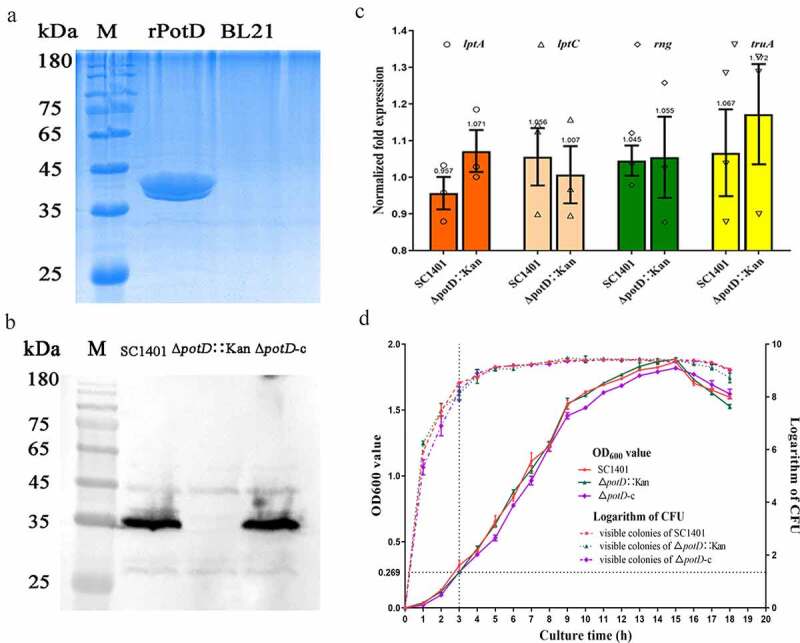
**The expression and purification of rPotD and confirmation of mutation, as well as growth curves of wild type, mutant and complementary strains**. (a) Purification of rPotD protein. Lane rPotD: purified rPotD protein (dialyzed, ultra-filtrated); lane BL21: results of purification of induced BL21 (pET-28a); M: protein molecular mass standard (Novoprotein). (b) Western blotting confirmation of *potD* mutant and its complemented strain. The lysates of the SC1401, Δ*potD*::Kan mutant and complemented strain Δ*potD*-c were detected using anti-PotD antibodies. The Δ*potD*-c strain displayed a similar band as that of the parent strain SC1401, while Δ*potD*::Kan strain did not. The first lane: *G. parasuis* wild-type strain SC1401; the second lane: Δ*potD*::Kan mutant; the third lane: complemented strain Δ*potD*-c; M: protein molecular mass standard (Novoprotein). (c) Transcription levels of the neighboring genes around the knock-in site for derivatives of *G. parasuis* strains SC1401 and Δ*potD*::Kan determined by RT-qPCR using 16S rRNA as an endogenous control. The bars represent changes relatively to 16S rRNA. The data represent the average results of triplicate experiments, with standard deviations indicated. (d) The growth curves of the *G. parasuis* SC1401 and its derivatives (solid lines indicate growth curve; dashed lines indicate CFU by plate counting method). Bacterial growth was monitored by measuring optical density at 600 nm and at each time point (from the early logarithmic phase to the decline phase along the whole growth curve). The visible colonies were determined from the number on TSA++ agar. Data points represent the mean values of three replicates, and error bars indicate standard deviations (SD)

rPotD protein was expressed as a fusion protein in its active form with both of its C-terminal and N-terminal 6× His tags, facilitating the purification process using a Ni affinity chromatography in vitro. The products were dialyzed, ultra-filtrated and analyzed by utilizing 12% SDS-PAGE, while the *E. coli* BL21 strain harboring the empty pET-28a(+) vector alone served as a negative control. One band with molecular mass of approximately 39 kDa corresponding to rPotD protein, plus its recombined tags, was obtained as expected (Figure 4a), indicating that the rPotD protein was successfully expressed in BL21(DE3) under the induction by 1 mM of IPTG for 5 h at 37°C. After endotoxin removal, the concentration of endotoxin was less than 0.1 EU per ml in the purified rPotD. The purified rPotD was subsequently used to vaccinate mice and treat macrophages Raw 264.7 and epithelial cells MLE-12 in the following study.

The anti-rPotD serum was prepared as previously described [Dai *et al*., 2019b]. Results showed that immunization with 0.1 mg of rPotD could sufficiently elicit a specific antibody titer up to 1 × 10^5^ on average, which could trigger both cellular and humoral immunity, facilitating our utilization of this antiserum to locate the native PotD in bacteria and to determine the successful construction of *potD* gene mutant and complemented strains in later studies.

## Construction of the *G. parasuis* Δ*potD*::Kan mutant and complemented strain Δ*potD*-c

The in-frame nonpolar *potD* mutant was constructed by naturally transforming the homologous fragments and uptake-signal sequences harboring suicide plasmid pK18-*potD* into a highly transformable recipient *G. parasuis* strain SC1401 in maximal competence-inducing conditions. The kanamycin-resistant transformants were preliminarily PCR identified using the primer set P3/P4 (*potD*-F/R, Table 2) to detect the deletion of *potD* gene in situ. Another primer set P9/P10 (Kan-F/R) was simultaneously applied to verify the presence of about 900-bp Kan^R^ cassette at the same locus of the deleted *potD* gene. For genetic complementation identification, pSF-c-*potD* sufficiently recombined into a locus on the chromosome just adjacent to the *ompA* gene in *G. parasuis*, which can be detected using primers P3/P4 and P19/P20 (Gm-F/R, 534 bp), confirming the representation of *potD* gene on the chromosome (gel electrophoretograms are not shown).

To preclude the possibility that the knock-in segment would affect the expression levels of flanking genes on the host DNA, we further performed qRT-PCR to check the transcriptional levels of four flanking genes (*lptA, lptC, rng* and *truA*) of the gene knock-out site on the chromosome of *G. parasuis*. As shown for the gene-deleted strain, none of the flanking genes were affected significantly when compared with those of wild type strain SC1401 (Figure 4b), indicating a nonpolarity effect.

To further determine the successful construction of *G. parasuis* mutant strain Δ*potD*::Kan and its gene-knock-in-based complemented strain Δ*potD*-c at the protein level, a routine WB analysis was used to detect the whole-cell extract of candidate *potD* mutant and its complemented strain. As depicted in Figure 4c, wild strain SC1401 and the complemented strain Δ*potD*-c that received pSF-c-*potD* engendered the same band at around 35 kDa, while Δ*potD*::Kan did not produce such a protein band. Taken together, these results are further evidence for the successful deletion/complementation of *potD* genes in strain SC1401.

## Genetic stability and growth characteristic of Δ*potD*::Kan Mutant

To preclude the possibility that the virulence attenuation or enhancement may result from suppressed or increased metabolic level of Δ*potD*::Kan, and that a reverse mutation might occur, we checked if *potD* deletion and knock-in were stable on the chromosome. Δ*potD*::Kan and Δ*potD*-c were serially passaged for 20 generations and PCR validated that gene deletion and recombination were stable on the chromosome of both derivatives. We demonstrated that the Δ*potD*::Kan strains did not show significant growth defects in vitro (p > 0.05) when compared with the wild-type SC1401 strains (Figure 4d). Further global alignment using whole-genome sequencing (WGS, GenBank No. NZ_CP015099.1) data confirmed that *potD* gene is an endogenous single-copied gene in *G. parasuis* SC1401, as shown by the successful and valid construct of *potD* gene mutant and complemented strains.

## Mutation of *potD* alters autosedimentation and biofilm formation of *G. parasuis* SC1401

**Figure 5.**
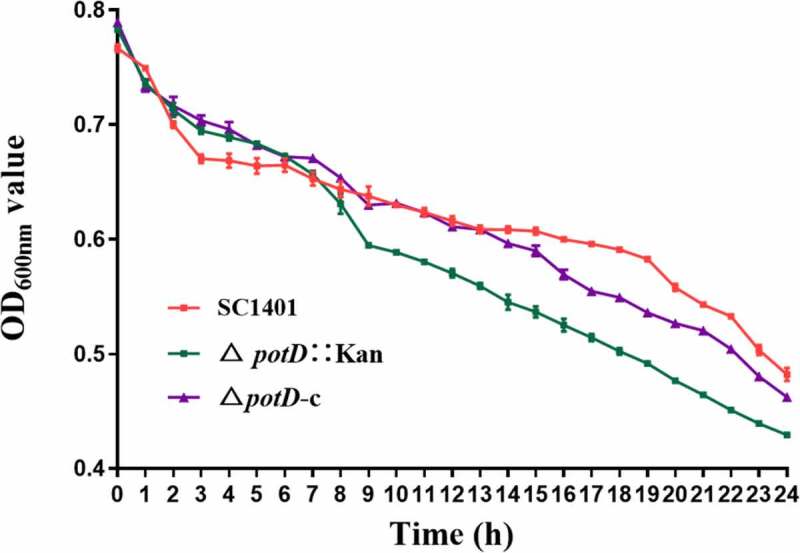
**Autosedimentation rates of SC1401 versus the Δ*potD*::Kan and Δ*potD*-c strains**. The cells were harvested and diluted in sterilized PBS to an OD_600nm_ of 0.8 and then allowed to remain static at 25°C. The OD_600nm_ of the suspensions was measured every 1 h for 24 h. The experiments were performed three times independently in triplicates. The means ± standard deviations from one representative experiment are shown

The ability to autoagglutinate (AA phenotype) is associated with virulence in some Gram-negative bacteria [Janda et al., 1987; Labandeira-Rey et al., 2010]. Compared with SC1401, the Δ*potD*::Kan strain showed an increased ability in autosedimentation (Figure 5), and complemented strain restored this phenotype to some extent, though it did not recover to the full level. We also noticed that there were more visible clustered bacterial adherence in the Δ*potD*::Kan culture when cultured to a late stage, suggesting that the amino acid changes introduced in Δ*potD*::Kan affected the strength of the interaction between the cells.

**Figure 6.**
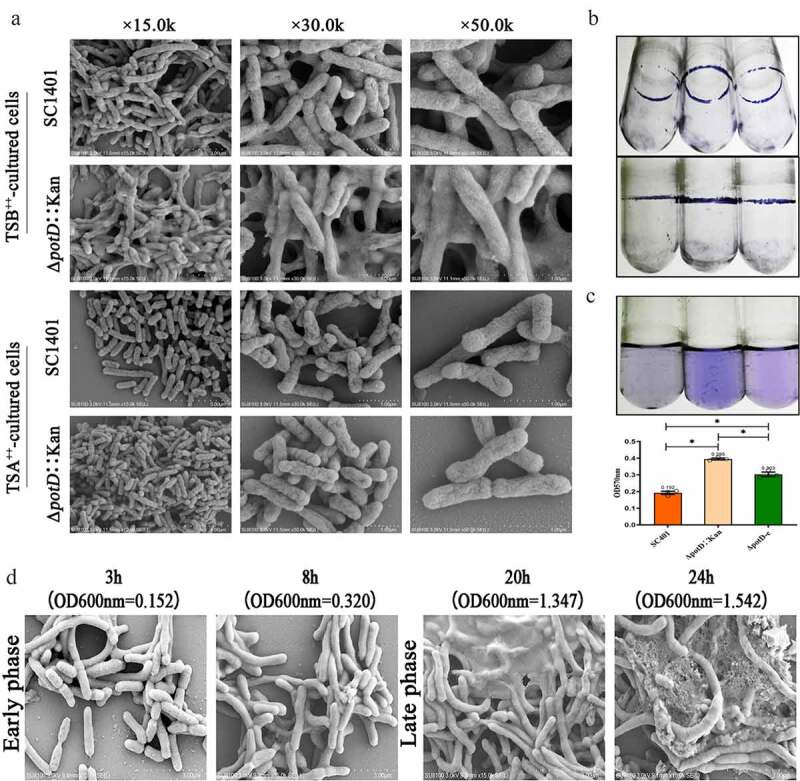
**Biofilm production of *G. parasuis* SC1401, Δ*potD*::Kan and Δ*potD*-c strains, and SEM of plate-cultured and broth-cultured bacteria**. The abilities of *G. parasuis* strains to form biofilm in TSB++-cultured conditions were quantified by the crystal violet incorporation assay. At the same time, Sections for statically cultured bacteria and suspended bacterium fluid (adherent and planktonic cells) were visualized using SEM. (a) Scanning electron microscopy analysis of the biofilms developed by *potD* gene knock-out in TSB++ – and TSA++-cultured bacteria. (b) Crystal violet stained biofilms formed at the air-liquid interface of glass tubes. Lane 1: *G. parasuis* SC1401; Lane 2: *G. parasuis* Δ*potD*::Kan; Lane 3: *G. parasuis* Δ*potD*-c. (c) Quantification of biofilm production by OD_570nm_ measurement. Lane 1: *G. parasuis* SC1401; Lane 2: *G. parasuis* Δ*potD*::Kan; Lane 3: *G. parasuis* Δ*potD*-c. Error bars represent the standard deviations of three independent experiments. (d) SEM observation of surface structures of the early and late culture of Δ*potD*::Kan (a time-dependent manner observation)

To further connect the phenotypes of autosedimentation and cellular matrix, we applied SEM and TEM in both TSA++ and TSB++-cultured bacteria in conjunction with the crystal violet-staining experiment of biofilm. In SEM, no obvious surface morphology differences could be identified between wild-type and *potD*-mutant strains when cultured on TSA++ for 20 h. Notably, however, Δ*potD*::Kan showed a significantly recognizable and abundant cellular matrix that partially encased bacteria, and the amount of the cellular matrix was much more than that outside the wild-type *G. parasuis* SC1401 (Figure 6a). This culture-condition-based cellular matrix’s diverse structure was then compared with the slow-growing-based biofilm formed by *G. parasuis* using a crystal violet staining experiment. Through crystal violet incorporation assay, we confirmed that biofilm was significantly augmented in Δ*potD*::Kan when compared with wild-type SC1401 and genetically complemented strain Δ*potD*-c (Figure 6b **and** Figure 6c). In light of these findings, we further observed the early and late phases of bacteria culture by SEM at four-time points. As depicted in Figure 6d, an apparent microscopic extracellular matrix increased with prolongation of culture time and growing stage, especially when Δ*potD*::Kan reached its stationary phase (OD_600nm_ = 1.347 and 1.542), suggesting that the formation of biofilm in Δ*potD*::Kan was a time-and – bacterial state-dependent or related event.

**Figure 7.**
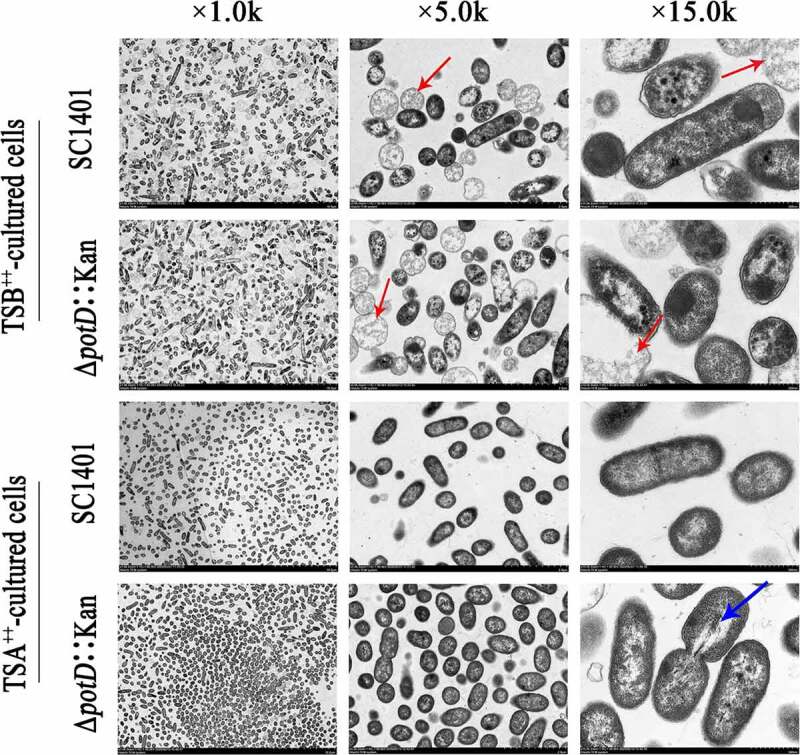
**Ultrastructures of plate-cultured and broth-cultured bacteria**. Sections for statically cultured bacteria and suspended bacterium fluid (adherent and planktonic cells) were visualized using transmission electron micrographs. TEM of statically cultured bacteria SC1401 and Δ*potD*::Kan showed several distinct differences in their cellular structures and morphotypes. Bacteria lysates were shown in red arrows, and a low electron density region was represented as a blue arrow. Different columns demonstrated three levels of magnification images

In TEM, cleaved and inflated cells or debris could be seen for both SC1401 and Δ*potD*::Kan strains after cultured for 20 h in TSB++ (Figure 7). In comparison, after both strains were grown on TSA++ for 20 h, we found Δ*potD*::Kan demonstrated conglobate morphology and there are lower electron density regions in the bacterial vertical axis of Δ*potD*::Kan. Collectively, these findings showed that TSB++-cultured bacteria alongside *potD*-mutation mediated biofilm that greatly differed from the wild-type bacteria-based biofilm. However, there is no *pgaABCD* operon or homologous genes identified in *G. parasuis* responsible for PGA (a bacterial exopolysaccharide that helps the host build biofilm matrices) synthesis, thus a more detailed molecular basis regarding biofilm formation in this species has yet to be elucidated.

## *potD* Mutant in two-tier environments demonstrates opposite oxidative pressure and SDS tolerance

**Figure 8.**
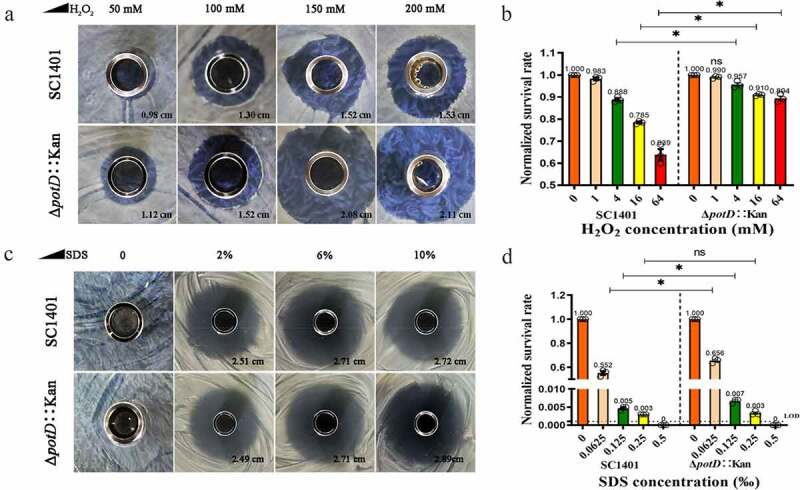
**Oxidative stress and anionic detergent SDS-resistance analysis based on inhibition ring test (the Oxford cup assay)**. (a) TSA++-cultured Δ*potD*::Kan became more sensitive to H_2_O_2_, but biofilm production in Δ*potD*::Kan greatly increases H_2_O_2_ resistance (b). (d) Δ*potD*::Kan cultured in TSB++ demonstrated a significantly higher survival rate under the low concentrations of SDS (less than 0.0125‰) than wild-type SC1401. The asterisk * indicates significance at a p < 0.05 level. These experiments were repeated three times. Each error bar represents one standard deviation from the mean. LOD: limit of detection

Since Δ*potD*::Kan demonstrated different morphotypes in TSA++ – and TSB++-culture conditions, to further examine the consequences of biofilm production in the GD infection process, we analyzed the ability of biofilm-producing cells to cope with the presence of different concentrations (50 mM to 200 mM) of H_2_O_2_. As shown in Figure 8a, besides demonstrating more visible lower electron density regions within the bacteria by microscopy, Δ*potD*::Kan exhibited relatively higher susceptibility to oxidative stress when grown on the surface of TSA++ in the form of bacteria lawn. Significant differences existed between groups under different oxidant concentrations. However, the opposite situation occurs when bacteria were grown in TSB++ and challenged with more than 4 mM of H_2_O_2_ (Figure 8b). For suspended bacterium fluid, H_2_O_2_ with a concentration less than 1 mM was inefficient to conferring bacteriostatic or bactericidal effects. At the same time, Δ*potD*::Kan showed a higher level of H_2_O_2_ tolerance, especially at 64 mM (p-value = 7.004E-04) levels. We concluded from this comparison that the broth-cultured Δ*potD*::Kan provoked a higher tolerance of extracellular oxidative stress. To determine if the observed H_2_O_2_ sensitivity alteration mediated by biofilm was common to other membrane-active agents, we tested the sensitivity of these strains to the anionic detergent SDS. As observed, both bacteria could form a similar diameter of the inhibitory zone under different SDS concentrations when bacteria were cultured on TSA++, indicating no anionic detergent-resistant substances were produced in this case (Figure 8c). However, Δ*potD*::Kan cultured in TSB++ demonstrated a significantly higher survival rate under low concentration of SDS (less than 0.0125‰) than wild-type SC1401 with the same culture condition, despite the fact that the higher concentration of SDS could eliminate this biofilm-based anionic detergent-resistance (Figure 8d). Altogether, these diverse effects indicated that the increased biofilm triggered by the deletion of *potD* under broth-cultured condition caused a significant enhancement in H_2_O_2_/oxidative stress resistance in *G. parasuis* and suggested that this positive effect on resistance might be generalizable to other detergents.

## Planktonic Δ*potD*::Kan demonstrated decreased adherence and invasion abilities

To further delineate the effects of the *G. parasuis potD* gene on host cell interactions, swine-sourced PK-15, and the mouse-sourced MLE-12 cell lines were incubated with planktonic wild-type, mutant, and complemented strains (non-biofilm bacteria) to compare the adherence and invasion abilities.

**Figure 9.**
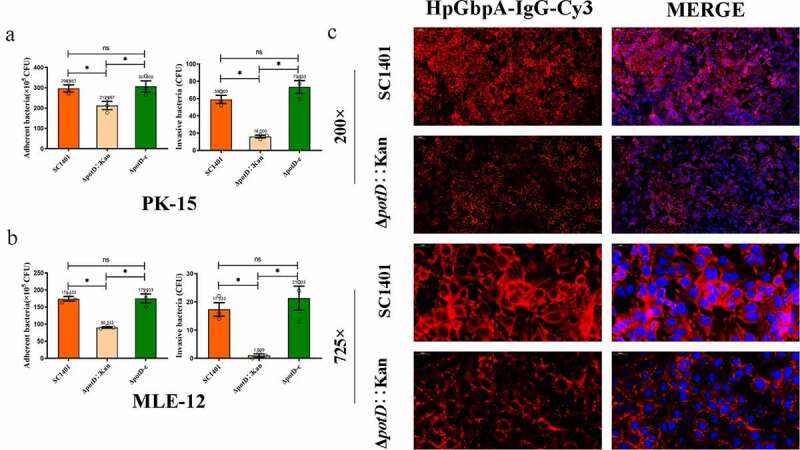
**Adherence and invasion abilities of *G. parasuis* to swine and mouse cell lines**. (a) Adherence (left histogram) and invasion (right histogram) to porcine kidney cell line PK-15. (b) Adherence (left) and invasion (right) to murine lung alveolar epithelial cell line MLE-12. The experiments were performed three times independently in triplicates. Error bars represent the standard errors from three independent experiments. The asterisk * indicates significance at a p < 0.05 level. These experiments were repeated three times. Each error bar represents one standard deviation from the mean. (c) Indirect immunofluorescence (IIF) analysis of adherent *potD* mutant to MLE-12. IIF assays were conducted to detect the distribution of bacteria by mouse-derived HpGbpA polyclonal antibody (1:500 dilution) and Cy3-conjugated goat-anti-mouse IgG (Servicebio)

As illustrated in Figure 9a **and** Figure 9b, when infection was performed at a multiplicity of infection (MOI) of 10, the adhered bacteria of mutant strain Δ*potD*::Kan was 212.667 × 10^5^ CFU/well on PK-15 cell surface and 90.333 × 10^5^ CFU/well on MLE-12 cells, which were significantly decreased in comparison with that of wild-type strain SC1401 (296.667 × 10^5^ CFU/well on PK-15 cells and 174.333 × 10^5^ CFU/well on MLE-12 cells). There was, therefore, significantly less invasion by the Δ*potD*::Kan mutant than the wild-type SC1401 strain on both cell lines (p < 0.05). Moreover, this result of reduced adhesion capacity of Δ*potD*::Kan to cell lines MLE-12 fitted a more visual observation using IIF assay that less relative fluorescence intensity (RFI) was found on the cell surface (Figure 9c). The adhesion and invasion levels were fully recovered in the complemented Δ*potD*-c strain. The results uncovered that *potD* affects the ability of the bacteria to interact with the two host epithelial cell lines PK-15 and MLE-12.

## PotD triggers the elevation of intracellular ROS in raw264.7 and animal

**Figure 10.**
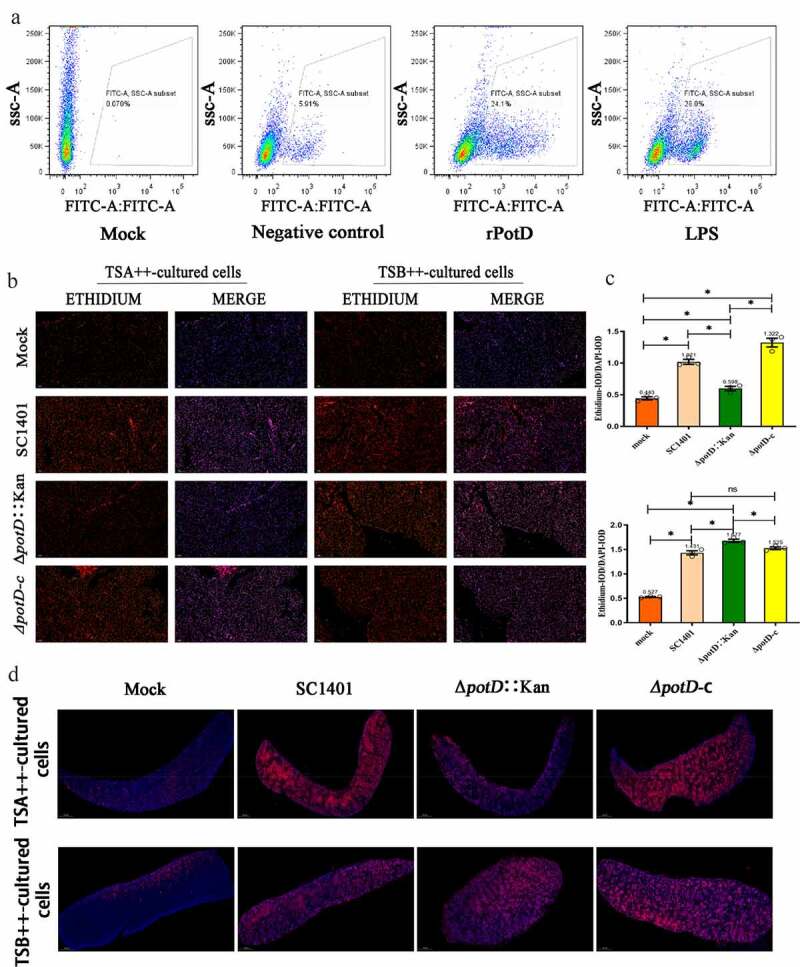
**The levels of ROS in cell model and animal model evaluated by FCM and IFA**. Cell-permeable fluorescent dye dichlorodihydrofluorescein diacetate (DCFH-DA) or dihydroethidium (DHE) can be oxidized to their corresponding oxide by intracellular ROS, which could integrate into chromosomal DNA to produce fluorescence, thus facilitating our evaluation of intracellular levels of ROS in cell model using FCM and in animals using IFA. (a) FCM analysis of intracellular level of ROS triggered by rPotD (EX WL: 488 nm, EM WL: 525 nm, a FITC-range monitoring scope). Frozen sections and IFA for (b) myocardia and (d) spleen tissues of the mice challenged with wild-type *G. parasuis* SC1401 and its derivatives Δ*potD*::Kan, and Δ*potD*-c after 4 dpi. (c) Statistical results for IOD of ROS levels in myocardia from mice challenged by *G. parasuis*. Relative values of IOD_R_ were calculated by the ratio of the IOD of fluorescence intensity (IOD) and DAPI (IOD_D_). The asterisk * indicates significance at a p < 0.05 level. These experiments were repeated three times. Each error bar represents one standard deviation from the mean

Intracellular levels of ROS were evaluated at both cellular and tissular levels. In the FCM assay, we used the fluorescence probe DCFH-DA (20 μM) to treat the mouse monocyte-macrophage leukemia cell line RAW 264.7. Only low background noise presented in the mock group, and only 5.91% of cell population input was sorted as ROS-positive, while this percentage significantly augmented after cells had been treated with endotoxin-deprived rPotD (24.1%), a figure slightly lower than that of cells treated with LPS (28.0%), serving as a positive control (Figure 10A). In IIF, there was also only low level of visible ethylene oxide observed in the mock group (mice received no challenge). However, in group of mice challenged by TSA++-pre-cultured bacteria, Δ*potD*::Kan induced a conspicuously lower level of ROS in myocardia evaluated by RFI (0.598 vs. 1.021) (Figure 10B **and** Figure 10c). The opposite result occurred after mice were challenged by TSB++-cultured Δ*potD*::Kan when compared with parent strain or complemented strain (1.677 vs. 1.431). A similar result was also visualized from the full field scans of spleen tissues shown in Figure 10D. This observation fitted our finding that the mutant bacteria under former culture conditions engendered more sensibility against H_2_O_2_, while TSB++-cultured Δ*potD*::Kan became more resistant to oxidative stress in vitro. Our results from three levels-in vitro test (oxford cup assay), the cellular and animal experiments, jointly proved that non-biofilm culture condition on TSA++ resulted in lower hyperoxic lung injury by *potD* knock-out in *G. parasuis*, but this negative effect could be overcome by the production of biofilm when bacteria were pre-cultured in TSB++.

## PotD is not involved in resistance to complement-mediated killing

Serum bactericidal assay (SBA) was performed to assess the survival rate of the Δ*potD*::Kan strain in 10%, 20%, and 50% porcine sera. Compared with SC1401, the Δ*potD*::Kan strain showed no obvious significant altered sensitivity to pig serum (P > 0.05) (**Figure S2**), with both genetically derived Δ*potD*::Kan and Δ*potD*-c strains and wild-type SC1401 having survival rates ranging from 12.03%-12.82% in 50% porcine serum. The results demonstrated that PotD protein is not involved in resistance to complement-mediated killing to planktonic bacteria in the logarithmic phase.

## Two different pathologic manifestations

As different culture conditions resulted in disparate biofilm formation, diverse stress resistance (oxidation lesion and anionic detergent), as well as opposite reactive oxygen induction capacities in both cellular and tissular models with regard to the same knock-out construct of Δ*potD*::Kan, we further dissected their pathologic manifestations in mice and found some interesting results as follows:

## Attenuation of the TSA++-pre-cultured Δ*potD*::Kan Mutant in SPF BALB/c Mice

**Figure 11.**
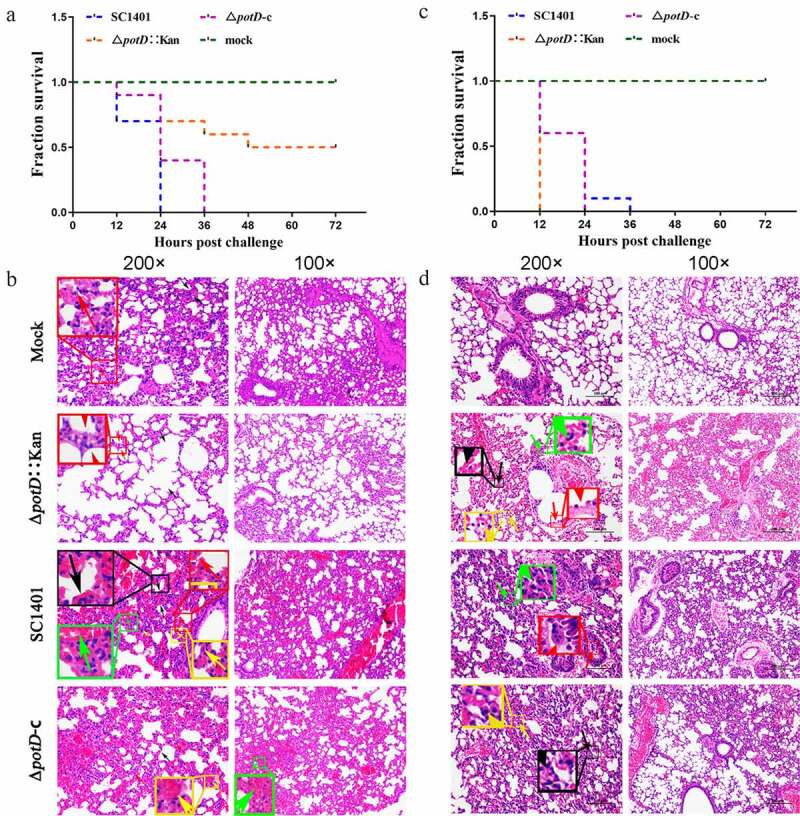
**The survival rate of mice challenged with a lethal dose (1.3 × 10^9^ CFU/mouse) of *G. parasuis* and histopathologic analysis of lungs (200×/100×)**. (a) In plate-culture, wild-type SC1401 and gene complemented strain Δ*potD*-c triggered 100% (10/10) death in mice model within 36 h post-injection. Δ*potD*::Kan mutant induced only a 50% (5/10) death rate in mice within a 72 h-period . (c) However, all broth-cultured bacteria could cause death in all challenged mice within 36 h. Lung tissues were harvested from mice in different groups after 4 dpi and used for HE staining and histopathological assay. (b) Lung tissues of mice challenged with plate-cultured bacteria. **Mock group** (Mock): slight inflammatory cell infiltration (red arrow). ***potD* mutant group** (Δ*potD*::Kan): thickening alveolar walls (red arrow). **Wild-type group** (SC1401): thickening alveolar walls and decreased number of alveoli (black arrow); capillary congestion (red arrow); neutrophil-based inflammatory cells infiltration on the alveolar walls (yellow arrow); red blood cells filled in small blood vessels (green arrow). ***potD* complemented group** (Δ*potD*-c): red blood cells congestion in small vessels (yellow arrow); hemorrhage and red blood cells within alveoli (green arrow). (d) Lung tissues of mice challenged with broth-cultured bacteria. **Mock group** (Mock): no visible pathological lesion. ***potD* mutant group** (Δ*potD*::Kan): loss of bronchial epithelial cells (red arrow); alveolar space stenosis (green arrow); scattered inflammatory cells infiltration along the alveolar walls (yellow arrow); congestion in extensive pulmonary vascular and alveolar wall capillaries (black arrow). **Wild-type group** (SC1401): a few epithelial cells could be seen in some tissues in the bronchioles (red arrow); slightly thickening alveolar walls and alveolar stenosis over a large area (green arrow); scattered inflammatory cells infiltration along the alveolar walls filled the entire field of vision. ***potD* complemented group** (Δ*potD*-c): thickening alveolar walls and alveolar space stenosis (black arrow); inflammatory cells infiltration along the alveolar walls (yellow arrow)

We performed animal infection studies to validate the contribution of PotD protein to bacterial virulence in vivo. As depicted in Figure 11A, Δ*potD*::Kan mutant engendered only 50% (5/10) of death in mice using an injection dose of 1.3 × 10^9^ CFU/mouse (0.5 ml) within a 72 h-period observation. In comparison, wild-type and gene complemented strains triggered rapid death in mice model within 36 h (100% death, 10/10). We found mice in the SC1401 and Δ*potD*-c groups presented severe clinical symptoms, such as weight loss, arthrocele, hunched posture, rough coat, lethargy, and shivering or fur loss, through clinical sign monitoring. Most mice scored up to 5–7 within 24 h post-infection using the murine activity scores system, while most mice in Δ*potD*::Kan scored 3–4 or less within 48–60 h. All hosts with nonlethal infections recovered as time prolonged. No anomalies were observed in the mock group throughout the entire bacteria-challenge experiments.

Lung and spleen tissues of hosts were harvested for further histological pathological observation using H&E staining. As shown in Figure 11B **and S3**, pathological lesions were present in all treated groups. However, compared with the mutant group, mice in wild-type and complemented gene groups showed increased thickening of alveolar walls and significantly decreased number of alveoli. The pulmonary bronchioles in both groups were filled with cellular exudate, which chiefly consisted of neutrophils and alveolar epithelial cells. There was more congestion in small blood vessels and blood capillaries and more substantial neutrophil-based inflammatory cells infiltration on the alveolar walls. Portions of the parenchyma were necrotic or collapsed. However, the mice in the Δ*potD*::Kan group demonstrated less severe pathological changes, most of which appeared as alveolar expansion, while the overall alveolar structure was easily visible (Figure 11B). In spleen tissues, the mice in wild-type and mutant groups displayed dilatation of blood sinus, hemolysis, macrophage reactions, etc. White pulp lymphocytes were more abundant and closely arranged. A small number of lymphocytes were necrotic in the white pulp, and the nuclei were deflated, fragmented, or dissolved. A larger number of red blood cells could be seen in the red pulp of the infected groups than in the mock group. However, Δ*potD*::Kan still caused severe lymphocytic necrosis in the white pulp, with many deflated, fragmented, or dissolved nuclei (**Figure S3**). No sign of injury was observed in the mock group. All these data together showed that while the *potD* mutant could still cause pathological lesions, the symptoms and pathological changes of hosts were less pronounced in *potD* mutant group than mice in wild-type and complemented gene groups, indicating that the deletion of *potD* gene led to a significant attenuation of TSA++-pre-cultured *G. parasuis* in the mouse model with regard to the degrees of pathological damage.

## TSB++-pre-cultured Δ*potD*::Kan mutant recovered its phenomenon of attenuation of Virulence

When SPF BALB/c mice were challenged with TSB++-pre-cultured bacteria, however, Δ*potD*::Kan mutant engendered 100% (10/10) of death in mice using an injection dose of 1.3 × 10^9^ CFU/mouse (0.5 ml) within a 12 h-period observation (Figure 11C), demonstrating a similar virulence level to wild-type and gene complemented strains which also triggered rapid death in mouse model (100% death, 10/10) within 24–36 h. Some severe pathological symptoms and inflammatory injury in the group of mice challenged by Δ*potD*::Kan appeared as bronchial epithelial cells disappearing, alveolar space stenosis, scattered inflammatory cells infiltration along the alveolar walls, and congestion in extensive pulmonary vascular and alveolar wall capillaries. (Figure 11D). This phenomenon of regaining virulence may partly be ascribable to a specific resistance of biofilm-forming bacteria to host immune clearance or multifactorial sterilization pressure. The details have yet to be elucidated.

## Inflammation and apoptosis evaluation in lung and spleen

**Figure 12.**
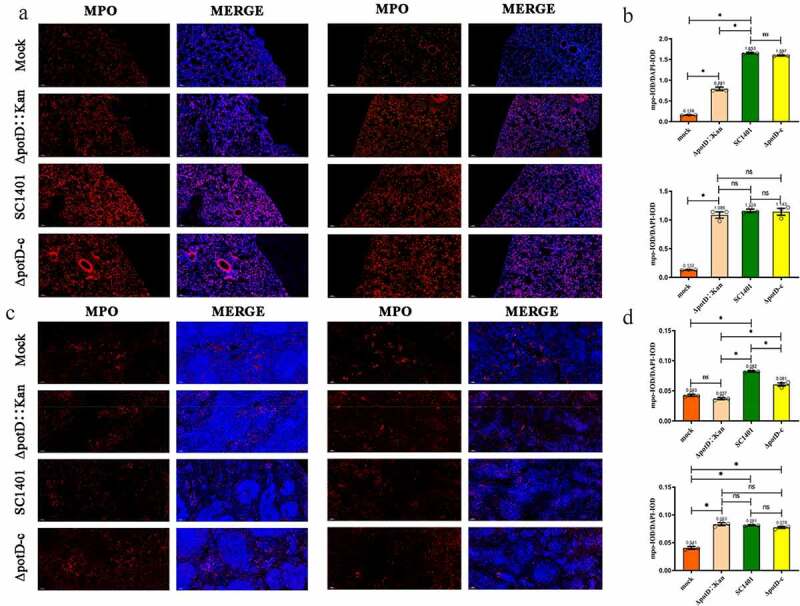
**Immunofluorescence (IF) assay for the level of myeloperoxidase (MPO) in lung and spleen tissues**. Lung and spleen tissues were harvested from mice in different groups after 4 dpi and used for FFPE treatment, pathological slice preparation and IF assay for myeloperoxidase (MPO). (a) IF analysis of lung tissue slides (100×). (b) Statistical analysis of IOD of myeloid cells (Biomarker: MPO) in mice lungs. Upper panel: group of mice challenged with TSA++-cultured bacteria; lower panel: group of mice challenged with TSB++-cultured bacteria. (c) IF analysis of spleen tissue slides (100×). (d) Statistical analysis of IOD of myeloid cells in mice spleen. Upper panel: group of mice challenged with TSA++-cultured bacteria; lower panel: a group of mice challenged with TSB++-cultured bacteria. Relative values of IOD_R_ were calculated by the ratio of the IOD of CY3-fluorescence intensity (IOD_C_) and DAPI (IOD_D_). The experiments were performed three times independently in triplicates. Error bars represent the standard errors from three independent experiments

As the IF analysis results of neutrophils showed, after 4 dpi, IOD in lung tissues of mice in all treated groups was significantly higher than those of the mock group (p < 0.05), indicating elevated MPO activity. However, in TSA++pre-cultured group, the IOD_R_ of SC1401 (average IOD_R_: 1.653) and complemented groups (average IOD_R_: 1.597) were significantly higher than that for the Δ*potD*::Kan mutant group (average IOD_R_: 0.791), indicating that *potD* mutant elicited less severe secondary inflammatory reactions and the release of toxins caused by *G. parasuis* colonization in the lung tissue after mouse challenge (Figure 12 **A and B**). In spleens, MPO levels in Δ*potD*::Kan group were insignificantly different (p > 0.05) compared with those observed in the mock group (Figure 12 **C and D**), and even much lower than that in the wild-type SC1401 and complemented groups, further supporting the conclusions in the H&E assay, where we found *potD* mutant strain engendered less severe inflammatory lesions in lungs and spleens. However, the opposite results occurred when bacteria had been pre-cultured in TSB++, which can be exemplified by their close IOD_R_ value (1.159 vs. 1.086 for SC1401 and Δ*potD*::Kan, respectively) a fact supporting our findings in H&E assay, in which these two disparate growing environments gave rise to different virulence phenotypes for the same knock-out of *potD* gene in *G. parasuis*.

**Figure 13.**
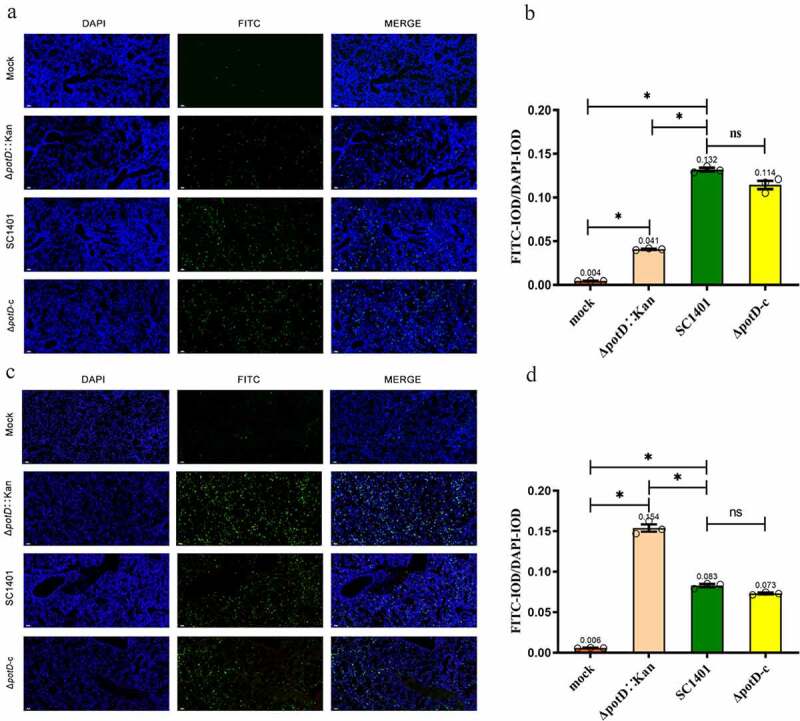
**Apoptosis assay for the level of pathological lesion in lung tissues (TUNEL staining)**. Lung tissues were harvested from mice in different groups after 4 dpi, and used for FFPE treatment, pathological slice preparation and IF assay for apoptosis. (a) IF analysis for the group of mice challenged with TSA++-cultured bacteria (100×). (b) Statistical analysis of IOD of myeloid cells (Biomarker: mpo) for figure A. (c) IF analysis for the group of mice challenged with TSB++-cultured bacteria (100×). (d) Statistical analysis of IOD of myeloid cells for figure C. Relative values of IOD_R_ were calculated by the ratio of the IOD of FITC-fluorescence intensity (IOD_F_) and DAPI (IOD_D_)

Quantification of bacterial apoptosis-inducing capacity in lungs using TUNEL staining confirmed pathological lesions directly in the level of cell death. The IOD of FITC-labeled fluorescent secondary antibodies augmented noticeably in all treated groups (Figure 13), as observed in the H&E assay. As expected, for TSA++pre-cultured group, the average value of IOD_R_ of Δ*potD*::Kan was merely one-third of that caused by wild-type strain when using their corresponding IOD_D_ of their viable cells as a reference. The average IOD_R_ in Δ*potD*::Kan was 0.041, while IOD_R_ in the wild-type SC401 group came to 0.132. The complemented gene strain showed a certain degree of restoration in cell-damaging capacity (average IOD_R_ equaled to 0.114). However, in the TSB++pre-cultured group, the average value of IOD_R_ of Δ*potD*::Kan was 0.154, which is much higher than that in the wild-type strain group (0.083) or gene complemented group (0.073), indicating a more severe apoptosis level. These results were consistent with the findings and scoring for the visual presence of nucleolysis, cell shrinkage, and the formation of apoptotic bodies. The linkage between the *potD* mutant and the particular apoptosis-mediating pathway is uncertain at the present time.

## Discussion

*Haemophilus parasuis*, or *Glaesserella parasuis* (*G. parasuis*) according to the new taxonomic reclassification, is usually a benign swine commensal in the upper respiratory tract, but may cause severe vascular lesions and multi-organ dysfunction, eliciting serious economic losses annually worldwide [Lin et al., 2019; Wang et al., 2017]. Several virulence factors have been reported; these include outer membrane proteins (OMPs) [Varela et al., 2014], capsular polysaccharide (CPS) [Perry et al., 2013], pilus, fimbriae, transferrin‐binding protein trimeric autotransporter (VtaA) [Costa-Hurtado et al., 2012], two-component signal transduction system (TCSTS; ArcA) [del Rio et al., 2006; Ding *et al*., 2016; Martinez et al., 2010], enzyme [Lichtensteiger and Vimr, 2003], and bacterial toxin (Cytolethal distending toxin, CDT) [Li et al., 2017], et al. Inflammation in porcine lungs and joints are usually the clinical signs for GD. Commercial bacterins are often used to vaccinate swine against *G. parasuis*, but the deficiency in cross-reactivity make sole-serotype inactivated vaccine an ineffective means of protection [McCaig et al., 2016]. Understanding the molecular basis of pathogenicity is vital in the design and implementation of vaccine and targeted treatment strategies.

Although microorganisms can obtain polyamines through the subtle multiple *de novo* synthesis pathways, the cost-effective transport systems, such as PotABCD, or other putrescine-specific transporter systems PotF, PotH, PotI and PotG in *E. coli*, still occupy essential parts in polyamine accumulation [Furuchi *et al*., 1991]. These conclusions have been drawn from the observation of the construction of Δ*potD* and its polyamine-uptake capacity alteration using the radiolabeled polyamines through rapid filtration method [Pistocchi et al., 1993; Raksajit et al., 2006]. The removal of one transport element could significantly decrease the pool of available polyamines, or at least, possibly make the self-synthesis pathway engage as a compensatory supplement for a certain kind of polyamine, which may happen at the cost of energy consumption that brings about energy depletion of other pathways as a whole. On account of its multifunctional role of polyamines in organism, it is reasonable to speculate that the null mutation of this binding protein PotD in *G. parasuis* would affect the host from different physiological functions.

The crystallographic study of the PotD protein at an atomic resolution demonstrates the detailed structural mechanism of its specific substrate bias for spermidine, despite putrescine binding as a subordinate substrate [Furuchi *et al*., 1991; Sugiyama *et al*., 1996b]. In this study, we identified five conserved polyamine-binding residues in *G. parasuis* PotD amino acid sequence: Asp-Glu-Trp-Trp-Asp, which were proven to be crucial for polyamine binding through crystal structure assay and homology modeling analysis in *E. coli* and *Synechocystis* sp., therefore suggesting that *G. parasuis* PotD protein may still possess the capacity to bind polyamines and very likely favors spermidine over putrescine [Igarashi et al., 1999; Sugiyama *et al*., 1996b]. A lipoprotein signal peptide was detected in the PotD with a cleavage site between position A22 and N23, and TMHMM2.0 server demonstrated that PotD has no typical transmembrane helix, suggesting that the protein is transported as a precursor protein through the cell membrane, lipid-modified and retained at the inner membrane. This implied that this protein was more likely to be hydrophilic, which is consistent with the GRAVY value (−0.331) of PotD calculated by the ExPASy ProtParam tool. Further analysis with regard to subcellular location based on both bioinformatics analysis and immuno-electron microscope (IEM) technology revealed that this protein is located in periplasm, and this visible research method has never been previously reported in *G. parasuis* but was successfully applied in this study for the first time. Our findings fit most physicochemical structures of ABC transport system – a large and diverse family of proteins, in which substances (ions, amino acids, nucleotides, polysaccharides, peptides, and even proteins) bind to a specific receptor and are transported to membrane-anchoring channels with the aid of an ATPase that produces energy. Most substrates bind to ATP to form a dimer structure, which is also the case of PotD protein in *E. coli* (PDB ID: 1POY) [Sugiyama *et al*., 1996a]. This prompted us to explain our observation that rPotD could form a “double-decker structure band” in SDS-PAGE (Figure 4a). In a verification study, the purified proteins in polyacrylamide gel (PAG) were excised after SDS-PAGE and analyzed by matrix assisted laser desorption ionization–tandem time of flight mass spectrometry (MALDI-TOF/TOF) using a 5800 Proteomics analyzer (Applied Biosystems) as previously described [Zhang *et al*., 2016]. Both peptide sequences matched the predicted amino acid sequence of PotD, confirming that the smaller protein may suffer from partial degradation or a steric hindrance existed in gel electrophoresis due to a high concentration. At the same time, this two-tier structure was not presented in native PotD protein, mainly due to limited amounts in the natural host condition.

In addition to involvement in polyamine transport, several lines of evidence suggest that *potD* participates in pathogenesis in many bacteria [Pipkins et al., 2017; Shah et al., 2008; Ware et al., 2006; Zhu et al., 2017]. We found Δ*potD* displayed growth kinetics almost identical to those of wild-type SC1401 in the TSB++ medium, indicating that the *pot* operon is not necessary for *in vitro* growth of the *G. parasuis* within such environments. This observation is in accordance with a previous finding in *S. pneumoniae* and further confirms that the virulence attenuation does not result from decreased metabolic level of Δ*potD* bacteria [Ware *et al*., 2006]. However, as for why *potD* mutant strain does not show a severely delayed growth curve (for polyamines are essential in many cellular physiological processes as stated above), a presumable interpretation is that an alternative polyamine biosynthesis and/or transport pathway(s) may be present within *G. parasuis*, or an alternative substrate may exist in the same metabolic regulatory pathways, working as a supplementary or two-tier regulatory factor. In general, the phenomenon is different from what we observed in *A. pleuropneumoniae*, in which the growth of the Δ*potD2* mutant was significantly affected in the logarithmic phase [Zhu *et al*., 2017]. In the present study, we also verified that Δ*potD* could be stably passaged, allowing it to serve as a useful construct in studies aimed at determining whether *potD* is involved in polyamine transport and/or whether it is crucial for virulence development in *G. parasuis*.

Putrescine and spermidine are involved in modulating the synthesis of DNA, RNA, and protein and can bind them through electrostatic binding, thus regulating gene transcriptional level. Putrescine can restore the expression level of the virulence factor VirF in a gene mutant *Shigella*, thus recovering normal virulence levels seen with this bacterium [Barbagallo et al., 2011; Leuzzi et al., 2015]. Polyamines have been shown to protect *E. coli* from toxic effects of oxygen, which allows bacteria to survive under harsh environment, and indirectly contributes to bacterial pathogenesis [Chattopadhyay et al., 2003; Patel et al., 2006]. In the present study, we found the rPotD protein alone could actively trigger the elevation of intracellular ROS levels on both cellular and tissular basis. This phenomenon could not be restored by adding supplementary exogenous spermidine (data not shown), suggesting that direct transportation of polyamine from the environment, rather than intracellular *de novo* synthesis, plays a more common or essential role in acquiring such substances. Another interpretation is that endogenous polyamine may be at a deficient level that can hardly maintain the needs of normal physiological functions. We combine the discoveries in this study and our previous report that PotD is a pro-inflammatory factor to verify that the *G. parasuis* derivatives Δ*potD* mutant can lead to attenuation in bacterial virulence compared with the wild-type strain SC1401 and complemented strain Δ*potD*-c in the SPF BALB/c mouse model. The animal infection studies in the present study also substantiate our former finding that PotD is one of the differentially expressed proteins between the highly virulent *G. parasuis* strain SC-1 (serotype 4; isolated from the lung of a diseased pig) and hypovirulent strain SC105 (serotype 3; isolated from the nasal swab of a healthy pig) [Zhang *et al*., 2014a]. TSA++-pre-cultured Δ*potD* mutant elicited significantly lower murine activity scores, lower rates of lethal infections, and relatively mild pathological lesions in the lungs and spleens of the challenged host. IF analysis demonstrated that Δ*potD* mutant engendered a lower level of oxidative damage and less severe levels of inflammation and apoptosis as well. However, we concurrently noticed that this negative impact on full bacterial virulence was tightly associated with its culture condition. In TSB++, bacterial virulence evaluation could be affected by the formation of biofilm or extracellular matrix as it is a common substrate that has high correlation to host pressure resistance [Wilkinson et al., 2018]. There might be other factors with regard to culture conditions, which can be exemplified by quorum sensing (QS), starvation or limitation in nutrients result from rapid growth, metabolites, and TCSTS. In light of these findings, we deem that a simple gene null knock-out may not be enough in evaluating virulence factors in the bacterial genome as a whole. Therefore, comprehensive influence factors, such as biofilm-formation, should be taken into account. Here we present a few lines of evidence indicating the importance of culture condition for bacterial virulence evaluation.

## Conclusions

Taken together, one of the primary findings to emerge from this study is that the conserved polyamine transporter PotD, which is located in the periplasm, is essential for bacterial virulence in *G. parasuis* in a mouse model. Overall, these results corroborate the findings of some of the previous work that PotD has been shown to be a crucial gene for bacterial virulence in *G. parasuis*. However, its negative effect on full virulence levels is tightly influenced by culture conditions, and the details remain to be studied. Also, a key uncertainty is that the mouse model cannot reflect the exact infection process of *G. parasuis* in the natural host. Further studies should be performed to evaluate the virulence of these strains using a typical porcine model. Our findings could lay some foundation for further virulence study of *potD* and polyamine transportation in swine model and hopefully aid in enriching knowledge and determining the pathogenicity and subunit antigen in *G. parasuis*, enabling researchers to develop improved strategies for the control of *G. parasuis* infection in the future.

## Ethical approval and consent to participate

All animal protocols were strictly performed under the approval from the Institutional Animal Care and Use Committee of Sichuan Agricultural University, Sichuan, China (Approval Number SYSK川 2019198). The China Regulations for the Administration of Affairs Concerning Experimental Animals (1988), and the Guide for the Care and Use of Laboratory Animals of the Ministry of Science and Technology of the People’s Republic of China were followed.

## Supplementary Material

Supplemental Material
