# Supplementary material for: Deletion of Polyamine Transport Protein PotD Exacerbates Virulence in Glaesserella (Haemophilus) parasuis in the Form of Non-biofilm-generated Bacteria in a Murine Acute Infection Model
Source: Virulence. 2021 Feb 2;12(1):520–46. doi: 10.1080/21505594.2021.1878673 (PMC7872090; doi:10.1080/21505594.2021.1878673)
Supplement: Supplemental Material [file KVIR_A_1878673_SM8418.zip › supplementary/Figure S1docking.docx]

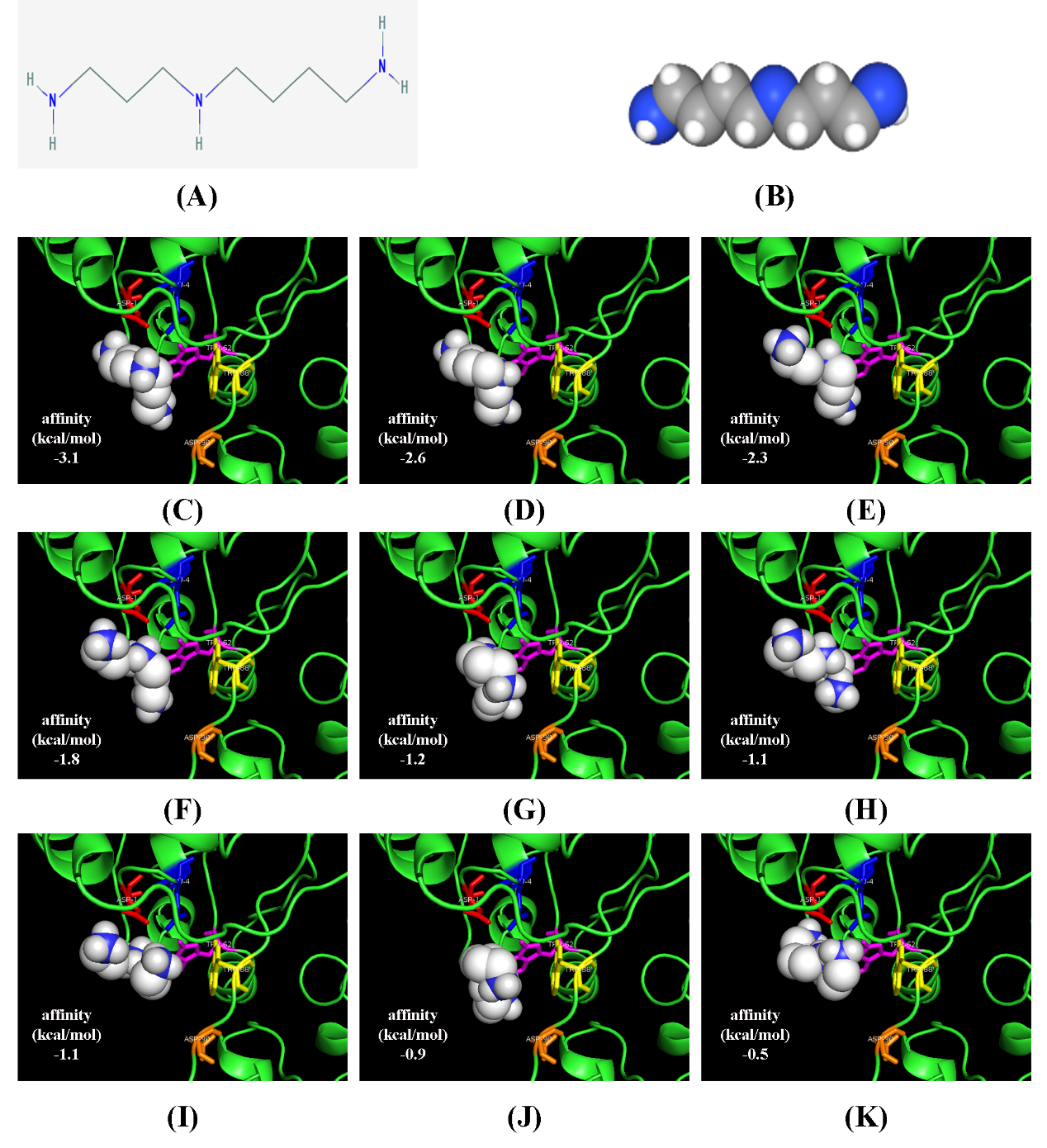


**Figure S1. Possible molecular docking of spermidine and spermidine-binding domain in *H. parasuis* PotD.** A set of nine models was created with the spermidine and its ligand in the active site. **(A)** Molecular formula and **(B)** pattern diagram of spermidine; **(C-K)** nine predicted models for molecular docking between spermidine and *H. parasuis* PotD.
