# Supplementary material for: Deletion of Polyamine Transport Protein PotD Exacerbates Virulence in Glaesserella (Haemophilus) parasuis in the Form of Non-biofilm-generated Bacteria in a Murine Acute Infection Model
Source: Virulence. 2021 Feb 2;12(1):520–46. doi: 10.1080/21505594.2021.1878673 (PMC7872090; doi:10.1080/21505594.2021.1878673)
Supplement: Supplemental Material [file KVIR_A_1878673_SM8418.zip › supplementary/Figure S2 serum.docx]

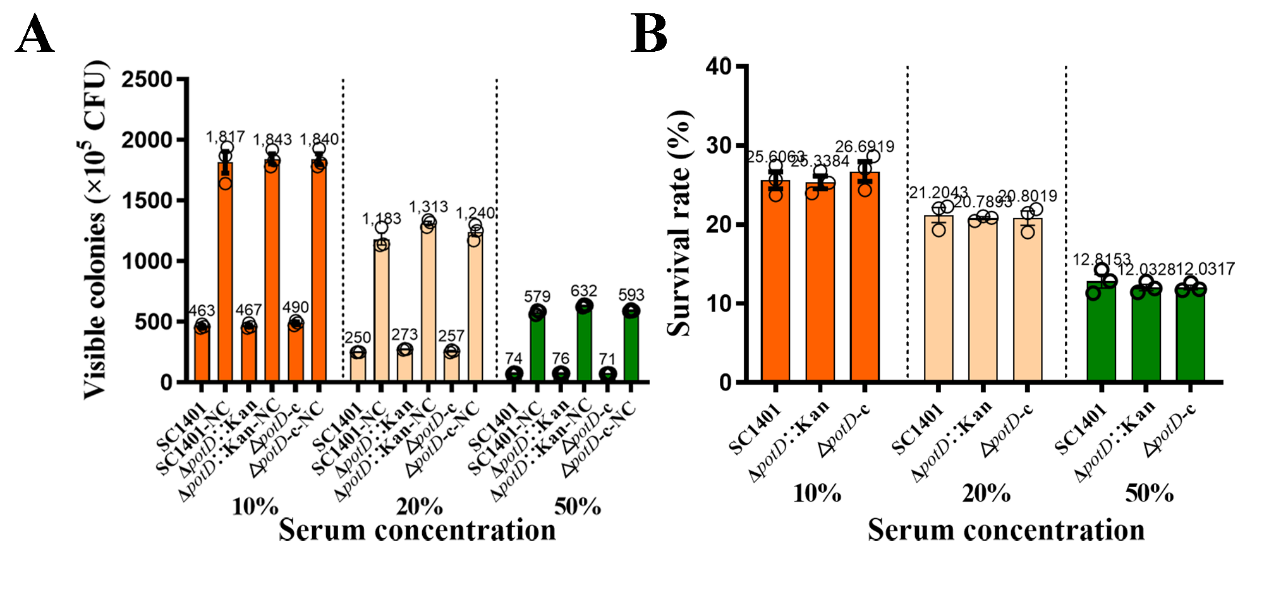


**Figure S2. Serum bactericidal assays.** (**A**) Visible colonies. (**B**) Survival rate. Percent survival was calculated as the ratio of the number of bacteria that survived in normal serum to the number that survived in heat-treated serum. The experiments were performed three times independently in triplicates. Error bars represent the standard errors from three independent experiments. The results for the Δ*potD*∷Kan, Δ*potD*-c and SC1401 have no significant difference (P>0.05).
