# Supplementary material for: Deletion of Polyamine Transport Protein PotD Exacerbates Virulence in Glaesserella (Haemophilus) parasuis in the Form of Non-biofilm-generated Bacteria in a Murine Acute Infection Model
Source: Virulence. 2021 Feb 2;12(1):520–46. doi: 10.1080/21505594.2021.1878673 (PMC7872090; doi:10.1080/21505594.2021.1878673)
Supplement: Supplemental Material [file KVIR_A_1878673_SM8418.zip › supplementary/Figure S3 spleen HE.docx]

**
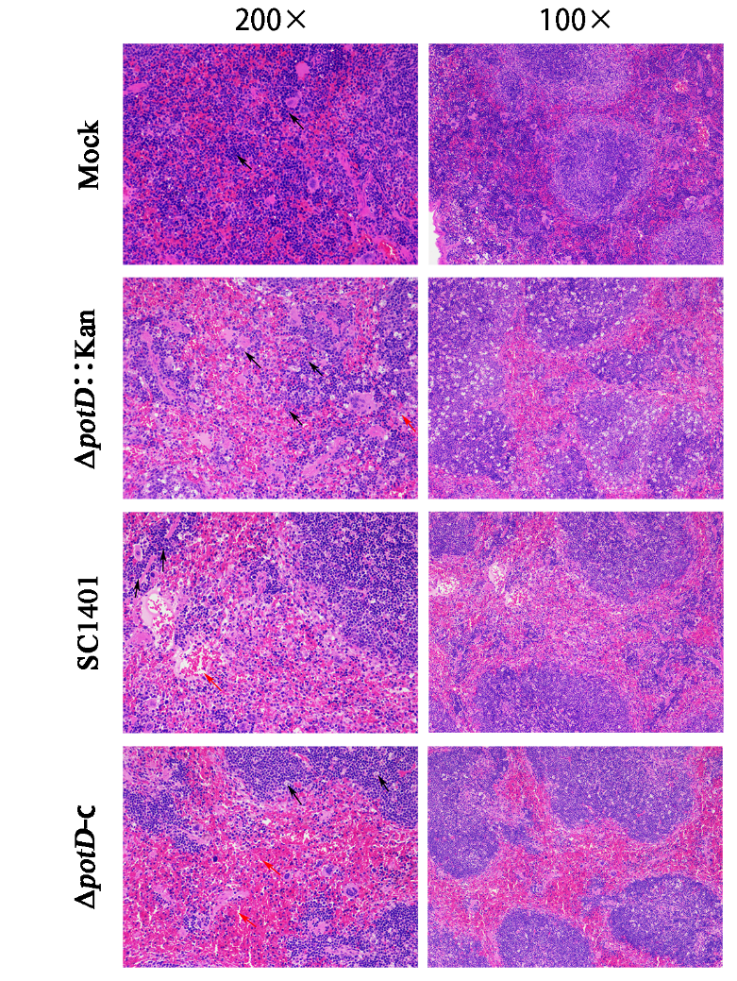
**

**Figure S3. Histopathologic analysis of mice spleens (200×/100×).** Spleen tissues were harvested from mice in different groups after 4 dpi, and used for HE staining and histopathological assay. **Mock group** (Mock): the boundaries of red and white pulps are clear; several extramedullary hematopoietic cells can be observed in red pulp area (black arrow). ***potD* mutant group** (Δ*potD*∷Kan): the boundaries of red and white pulps are clear, and the structure of white pulp is regular; there are many lymphocytic necrosis, karyopycnosis and fragmentation in the white pulp area (red arrow); a small number of extramedullary hematopoietic cells can be seen in the red pulp (black arrow). There are more red blood cells in the red pulp than in the normal spleen. **Wild-type group** (SC1401): the boundaries of red and white pulps are still clear, lymphocytes in white pulps are abundant and closely arranged, and a few extramedullary hematopoietic cells can be seen in the red pulps (black arrow); a small amount of splenic sinus dilatation could be seen (red arrow); there are more red blood cells in the red pulps than in the normal spleen. ***potD* complemented group** (Δ*potD*-c): the boundaries of red and white pulps are still clear. Lymphocytes in white pulps are abundant and closely arranged, and there are many lymphocytic necrosis, karyopycnosis, deep staining and fragmentation in the white pulp area (black arrow); a larger number of red blood cells could be seen in the red pulp compared with the normal spleens (red arrow).
