# Supplementary material for: Deletion of Polyamine Transport Protein PotD Exacerbates Virulence in Glaesserella (Haemophilus) parasuis in the Form of Non-biofilm-generated Bacteria in a Murine Acute Infection Model
Source: Virulence. 2021 Feb 2;12(1):520–46. doi: 10.1080/21505594.2021.1878673 (PMC7872090; doi:10.1080/21505594.2021.1878673)
Supplement: Supplemental Material [file KVIR_A_1878673_SM8418.zip › supplementary/Table S1 abbreviation of strain names.docx]

**Table S1. Abbreviation of strain names in this study**

| **Abbreviation** | **Nomenclature** |
| --- | --- |
| Hps | *Haemophilus parasuis* |
| Hin | *Haemophilus influenzae* |
| App | *Actinobacillus pleuropneumoniae* |
| Gan | *Gallibacterium anatis* |
| Aap | *Aggregatibacter aphrophilus* |
| Aac | *Aggregatibacter actinomycetemcomitans* |
| Afa | *Agrobacterium fabrum* |
| Hpa | *Haemophilus parainfluenzae* |
| Pmu | *Pasteurella multocida* |
| E. coli | *Escherichia coli* |
| Sbo | *Shigella boydii* |
| Vch | *Vibrio cholera* |
| Vfl | *Vibrio fluvialis* |
| Spn | *Streptococcus pneumoniae* |
| Ssu | *Streptococcus suis* |
| Smu | *Streptococcus mutans*  *Streptococcus mutans*  *Streptococcus mutans* |
| Ssa | *Streptococcus sanguinis* |
| Cdi | *Clostridioides difficile* |
| Nme | *Neisseria meningitidis* |
| Hdu | *Haemophilus ducreyi* |
